# Prostate Cancer Genomics: Recent Advances and the Prevailing Underrepresentation from Racial and Ethnic Minorities

**DOI:** 10.3390/ijms19041255

**Published:** 2018-04-22

**Authors:** Shyh-Han Tan, Gyorgy Petrovics, Shiv Srivastava

**Affiliations:** Center for Prostate Disease Research, Department of Surgery, Uniformed Services University of the Health Sciences and the Walter Reed National Military Medical Center, Rockville, MD 20852, USA; gpetrovics@cpdr.org (G.P.); ssrivastava@cpdr.org (S.S.)

**Keywords:** prostate cancer, cancer genomics, hereditary prostate cancer, cancer health disparities

## Abstract

Prostate cancer (CaP) is the most commonly diagnosed non-cutaneous cancer and the second leading cause of male cancer deaths in the United States. Among African American (AA) men, CaP is the most prevalent malignancy, with disproportionately higher incidence and mortality rates. Even after discounting the influence of socioeconomic factors, the effect of molecular and genetic factors on racial disparity of CaP is evident. Earlier studies on the molecular basis for CaP disparity have focused on the influence of heritable mutations and single-nucleotide polymorphisms (SNPs). Most CaP susceptibility alleles identified based on genome-wide association studies (GWAS) were common, low-penetrance variants. Germline CaP-associated mutations that are highly penetrant, such as those found in *HOXB13* and *BRCA2*, are usually rare. More recently, genomic studies enabled by Next-Gen Sequencing (NGS) technologies have focused on the identification of somatic mutations that contribute to CaP tumorigenesis. These studies confirmed the high prevalence of *ERG* gene fusions and *PTEN* deletions among Caucasian Americans and identified novel somatic alterations in *SPOP* and *FOXA1* genes in early stages of CaP. Individuals with African ancestry and other minorities are often underrepresented in these large-scale genomic studies, which are performed primarily using tumors from men of European ancestry. The insufficient number of specimens from AA men and other minority populations, together with the heterogeneity in the molecular etiology of CaP across populations, challenge the generalizability of findings from these projects. Efforts to close this gap by sequencing larger numbers of tumor specimens from more diverse populations, although still at an early stage, have discovered distinct genomic alterations. These research findings can have a direct impact on the diagnosis of CaP, the stratification of patients for treatment, and can help to address the disparity in incidence and mortality of CaP. This review examines the progress of understanding in CaP genetics and genomics and highlight the need to increase the representation from minority populations.

## 1. Introduction

Carcinoma of the prostate (CaP) is the second most frequently diagnosed cancer in men worldwide and the fifth leading cause of cancer death in men [1]. Among the 1.1 million new cases of CaP diagnosed, about two-thirds occur among men in developed countries, where 17% of the world’s male population resides. Despite the higher incidence in developed countries, CaP mortality is highest among men of African ancestry residing in the Caribbean and in Southern and Central Africa [1]. In the United States, CaP is the most common non-cutaneous cancer and the second leading cause of male cancer deaths in the US [2]. In comparison to Caucasian Americans (CA), African American (AA) men have 1.7 times higher incidence, and 2.4 times higher mortality rate [3]. Disparities in the diagnosis, treatment, and survival of CaP patients are often attributed to socio-economic status and access to healthcare [4,5]. However, even after adjusting for the effects of socio-economic factors, racial disparities in CaP incidence and mortality rates remain significant, suggesting for a greater contribution from molecular and genetic factors [6]. Among diverse ethnic populations, distinct interaction between genetic factors may contribute to the differential propensity for mutations in oncogenic drivers that contribute to the initiation and progression of aggressive cancers [7,8,9,10]. The stratification of patients based on the higher frequencies of specific oncogenic drivers that are associated with ancestry can lead to more effective treatments. For example, a higher prevalence of epidermal growth factor receptor (EGFR) mutation among female non-small-cell lung cancer (NSCLC) patients of East Asian descent who are non-smokers often results in dramatic responses to EGFR tyrosine kinase inhibitors, leading to a more favorable prognosis for overall survival [9,11]. 

The initial breakthrough in sequencing DNA by chain terminating method [12] opened a path of discovery that led to the Human Genome Project and the successful mapping of the human genome [13,14]. This milestone event transformed genetic and genomic discoveries by establishing a standard reference genome to which diseased samples can be compared, and thus enable the identification of putative genetic defects. The completion of the human genome also fostered the HapMap [15] and the 1000 Genomes projects [16], which produced extensive catalogs of human genetic variations that facilitated the study of multifactorial diseases, including cancer, using genome-wide association studies (GWAS) [17]. The progress in Next-generation sequencing technologies further accelerated the discovery of genomic alterations, allowing for the grouping of some cancers into subtypes, and placed personalized or precision medicine within reach of most cancer patients. Since the heterogeneity in the molecular basis of CaP across different ethnic or racial population groups is poorly understood, the under-representation of ethnic or racial minorities in most large scale genomic analyses of CaP will delay the discovery of unique genomic alterations and prevent the generalizability of findings from these studies [18]. This review will examine the progress in our understanding of the genetics and genomics of CaP, from the discovery of CaP susceptibility alleles, through identification of germline mutations, to recent developments in the detection of somatic gene alterations in localized and metastatic CaP genomes, in the context of existing CaP disparity between AA and CA men.

## 2. Assessing the Contribution of Mendelian Inheritance to Prostate Cancer Risk by Segregation and Linkage Analyses Studies

**Figure 1:**
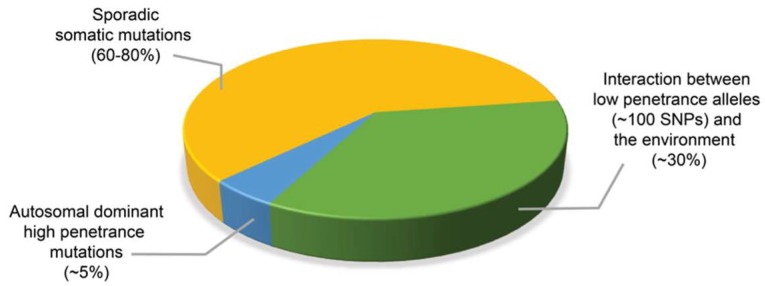
Approximate contribution of germline mutations, low penetrance alleles and sporadic somatic mutations to prostate cancer incidence. While most prostate cancer arise from sporadic somatic mutations, about 5% of prostate cancers develop from autosomal dominant highly penetrant germline mutations, and approximately 30% of cases of prostate cancer occur as a result of interaction between genes with low penetrance alleles and the environment.

Early quantitative genetic analyses of monozygotic and dizygotic twins estimated that germline mutations contribute approximately 42–58% to CaP risk, which is higher than for any other malignancies [19,20,21]. The relative risks for lethal CaP for men with one first-degree relative with CaP, selected from a National Cancer Institute (NCI) Surveillance, Epidemiology, and End Results (SEER) cancer registry in the state of Utah, was assessed at approximately 2.5, which increased to 5.3 for those with three or more affected first-degree relatives [22]. A population-based case control study of Caucasians, Blacks, and Asians reported that a family history of two or more affected first-degree relatives with CaP was associated with a higher relative risk of 9.7 in men of African ancestry, compared to 3.9 in Caucasians, and 1.6 in Asians [23]. Segregation analyses studies based on statistical methods were then used to establish models of predisposition to CaP linked to Mendelian patterns of inheritance, such as autosomal dominant, recessive, or X-linked [24,25,26,27]. Mendelian inheritance patterns are largely due to DNA alterations that give rise to pathogenic germline variants, or mutations in highly penetrant cancer predisposition genes such as in *HOXB13* [28,29] and *BRCA2* [30]. Mutations in these genes are responsible for hereditary cancer syndromes that are estimated to account for at least 5% of CaP [24,31,32] (Figure 1). However, since CaP is a relatively common disease, it is less likely to develop from a few rare inherited alleles with strong penetrance but more likely to have arisen from the interaction between multiple common alleles of low and intermediate penetrance and environmental factors, such as infection or inflammation, which are likely to account for a large proportion of familial prostate cancers [32,33,34,35]. The ability to further identify and assess the role of CaP susceptibility genes will benefit from advances in genome-wide association studies (GWAS) and incorporating family history of cancer in the genomic sequencing of germline DNA.

## 3. Identification of Prostate Cancer Susceptibility Loci by Genome-Wide Association Studies (GWAS)

GWAS are usually case-control studies that examine the entire genome for association between single-nucleotide polymorphisms (SNPs) with a trait or a disease. “Causal variants” are variants that confer a biological effect on the phenotype and are responsible for the signal in association studies [36]. Causal variants that have been validated in large matched control cohorts are useful for predicting clinical risk, but they account for a very small fraction of the underlying genetic contribution to CaP and fewer still are able to predict aggressiveness or survival [37,38]. While rare genetic variants have been shown to contribute significantly to increase risk of prostate and other cancers, they are often neglected or understudied because their low frequency precluded them from being tagged by conventional genome-wide genotyping arrays, which presents a drawback for GWAS efforts [39,40]. Larger populations of cases and controls are required to detect rare variants with lower minor allele frequencies. The high-fidelity of NGS has enabled the development of haplotype reference panels, established from low-coverage whole genome sequencing (WGS) datasets of large numbers of samples. This has allowed the imputation of genotypes of as low as 0.1% and extended the utility of GWAS to decode the allelic structure of cancer susceptibility [16,41,42,43]. Genotype imputation allows the prediction of genotypes that are not directly identified in the study sample. By combining a reference panel of individuals genotyped for a set of SNPs at a much higher density with a study sample derived from a genetically similar population but genotyped at only a subset of these sites, unobserved genotypes in the study sample can be imputed by extrapolation of allelic correlations measured in the reference panel [44].

### 3.1. The 8q24 Locus and Other Prostate Cancer Risk Alleles Associated with African Americans

Motivated by the aim to identify common germline variants contributing to the underlying risk of CaP, Amundadottir et al. [45] conducted a genome-wide linkage analysis in an Icelandic population. A variant at the 8q24 locus was found to contribute an attributable risk for CaP at approximately 8% increased risk among men of European ancestry, and 16% among men of African ancestry, suggesting a higher contribution to the incidence of CaP in the latter. Subsequent GWAS and an admixture mapping study replicated this association in different cohorts of AA men [46,47,48]. The rs1447295 variant at the 8q24 locus was found to be associated with both an earlier age of diagnosis and with an increased risk for CaP among AA men [49]. A later evaluation of seven SNPs that were sufficient to account for the admixture association signal identified three variants (rs16901979, rs7000448, and rs6983267) with the strongest associations and conferred significant risk for CaP in men of African ancestry [50]. Additional variants, including at least nine near 8q24 were later found to be independently associated risk for CaP among AA men [51]. A meta-analyses of risk variants on 8q24 confirmed that while there was a significant association of six variants with a higher risk of CaP for at least one race, the degree of association and frequency of the causative allele varied among men of different races [52] 

In the search for inherited variants that confer increased susceptibility to CaP among men of African descent, a case-control GWAS performed in over 3500 samples assembled from a consortium of CaP studies identified the rs7210100 SNP at 17q21, which was found to occur at 5% frequency in AA men, compared to <1% in other populations [53]. A meta-analysis of the *CYP17* gene polymorphism (rs743572) in men of African ancestry found an association of the SNP with 60% increased risk of CaP in sub-group of AA men [54]. In a search for risk alleles associated with CaP aggressiveness in AA men, Whitman and colleagues [55] genotyped six risk SNPs within 8q24, against clinical variables. Patients harboring the Broad11934905A allele were found to have a higher pathologic stage (pT3-4) and showed a trend toward earlier biochemical recurrence, compared to individuals with the wild type allele. More recently, Koboldt et al. [56] analyzed genomic DNA from 150 CA and 122 AA CaP patients and 300 race-matched controls by whole exome sequencing (WES), followed by targeted sequencing of 800 genes in 652 aggressive CaP patients and 752 controls, of both AA and CA men. A rare deleterious variant of *TET2* associated with aggressive disease was detected at 24.4% among AA patients compared with 9.6% of controls.

GWAS has identified more than 100 common variants that account for up to 38% of the risk of familial CaP [38,57,58,59] (Figure 1). As of December 2017, a search on the National Human Genome Research Institute (NHGRI) and European Molecular Biology Laboratory - European Bioinformatics Institute (EMBL-EBI) GWAS Catalog database (Available online: www.ebi.ac.uk/gwas), retrieved a total of 63 “prostate carcinoma” GWAS, which identified 587 associations with CaP, located at more than 400 different loci. While most GWAS were performed using cohorts of Caucasian populations, there were efforts to conduct studies that comprise of more diverse racial, ethnic or geographical populations, either in the initial samples [59,60,61], or in the replicate populations [46,62,63,64]. Studies that included men of AA ancestry in the initial samples or in the replicate samples has allowed the detection of CaP susceptibility variants associated with African ancestry [46,53,59,65,66,67,68,69,70]. GWAS conducted among men from different populations, such as Latino [60], South Asian [71], Japanese, [60,72], and Chinese [73,74,75] ancestries have further identified potential race-specific differences. A large contribution to this list comes from a meta-analysis of >10 million SNPs from GWAS in populations of European, African, Japanese, and Latino ancestry [59]. This combined analysis across racial groups identified 12 new susceptibility loci, of which seven were identified in the multi-ancestry analyses. An examination of the generalizability of 82 established CaP risk variants in 4500 CaP cases and equal number of controls of African ancestry found that 68 variants (83%) had effects that were directionally consistent in their association with CaP risk, indicating that common functional alleles are shared across populations [68]. Similar studies that combine the data across populations are beneficial as they not only have increased sample size and study power, but also form independent replication sample sets that promote the discovery of rare variants [46,64,66].

### 3.2. Potential Mechanisms Contributing to the Association with Prostate Cancer Risk

The realization that CaP risk alleles identified in GWAS studies are consistently located in introns or intergenic regions of DNA raised the question of possible mechanisms that may regulate the association with CaP risk. Whether the perceived association has a direct cause or is pathogenic for the disease may need to be experimentally tested [76]. One hypothesis proposed that the identified variant or SNP is in linkage disequilibrium with a yet to be identified causative variant that lies within a coding sequence [77]. Another premise suggested that the risk allele may disrupt a transcription factor binding site of a gene promoter and affect its expression [78]. The second model is exemplified by the rs10993994 SNP, found 5′ to the *MSMB* gene at 10q11 [79,80]. *MSMB* encodes for the prostate secreted seminal plasma protein, PSP94, the expression of which is downregulated in CaP cells [81,82]. The association of rs10993994:C > T variant with decreased PSP94 expression observed in radical prostatectomy derived tissue specimens [83] was confirmed by functional studies that mutated the rs10993994 allele [84]. Homozygous rs10993994 T-allele carriers were later found to have the most significant association with DNA double strand repair capacity in blood lymphocytes after ionizing irradiation, which suggests that besides regulating *MSMB*, the rs10993994 genotype may affect genes associated with DNA repair and apoptosis [85]. 

The gain of 8q24 region frequently occurs in prostate tumors, and it is associated with aggressive tumors, hormone independence, and poor prognosis [86]. However, there was no consistent correlation between copy number amplification with mRNA or protein expression [87,88]. Since the 8q24 locus lies within a 1.2 megabase region largely devoid of genes, the location of the *c-MYC* proto-oncogene towards its telomeric end has prompted searches for an association between 8q24 risk variants and *c-MYC* [89,90]. An evaluation of the association between six 8q24 risk variants and the transcription of multiple genes in prostatectomy specimens from CA and AA men found no association between the expression of these genes and risk allele status [89]. Wasserman et al. [90] found a risk variant (rs6983267) located in an enhancer within 8q24 that was transcribed in synchrony with *c*-*MYC* during prostate development and proposed that it may confer CaP risk by acting in an early event before tumorigenesis. The hypothesis that the 8q24 risk locus may act as an enhancer element by being in contact with *MYC* [91] was demonstrated by Ahmadiyeh et al. [92] through the detection of long-range chromatin loops by using the chromosome conformation capture (3C) assay. Further experiments using 3C or circularized chromosome conformation capture (4C) coupled with next-generation sequencing may offer clues to interaction between 8q24 variants and *c-MYC* expression and subsequent CaP initiation and progression [92,93,94].

## 4. Germline Mutations Associated with Hereditary Prostate Cancer

**Figure 2:**
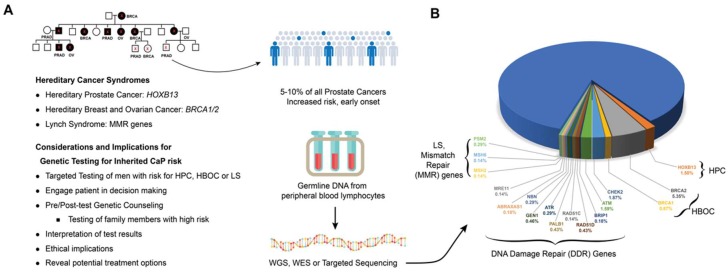
Hereditary cancer syndromes and prostate cancer. Men from families with hereditary cancer syndromes, including Hereditary Prostate Cancer (HPC), Hereditary Breast and Ovarian Cancer (HBOC) and Lynch Syndrome (LS), which are associated with defects in *HOXB13*, *BRCA1/2* and DNA Mismatch Repair genes, respectively, may benefit from genetic testing (**A**). Frequency of pathogenic germline alterations in DDR genes [30,100,101] and in genes associated with hereditary cancer syndromes (HPC [29,102], HBOC [103], and LS [104,105,106]) detected in metastatic prostate cancers (**B**).

The patterns of inheritance based on allele frequency and penetrance estimates established by segregation analyses were subsequently used in linkage analysis to localize chromosomal regions that are inherited together with the cancer causing gene [95]. Linkage analysis was used with positional cloning to successfully identify susceptibility genes in breast cancer, colon cancer, and renal cell carcinoma. Applying this strategy, several CaP susceptibility loci were successfully mapped: HPC1 at 1q23-25, PCAP at 1q42, CAPB at 1p36, linkage at 8p22–23, HPC2 at 17p11, and HPCX at Xq27 [32]. Candidate familial CaP susceptibility genes were identified for three of these linkages: *RNASEL* (mapping to the HPC1) [96], *ELAC2* (mapping to the HPC2) [97] and *MSR1* (mapping to 8p22–23 [98]. These CaP predisposition genes were found to harbor low- to moderate-penetrance alleles and affect pathways that influence prostate function. Highly penetrant mutations were also detected in genes that regulate critical steps in developmental program, such as *HOXB13* [28,29], DNA replication, such as *BRCA2* [30], DNA mismatch repair (MMR), such as *MSH2* [99], or DNA damage repair, such as *CHEK2* and *ATM* [100] (Figure 2).

### 4.1. ELAC2, RNASEL and MSR1

*ELAC2* was initially identified as a CaP susceptibility gene but subsequent studies that focused on its most common variants, S217L and A451T found both of these alleles to be of low penetrance [107,108]. A recent meta-analysis of 18 studies evaluating the these two alleles found both to be associated with increased CaP risk among CA and Asian men, but not among AA men [109]. However, a study of 188 sporadic and 55 familial AA CaP cases detected an association between the S217L allele and increase CaP risk among sporadic cases [110]. 

The role of *RNASEL* gene variation and its influence on CaP susceptibility has been controversial. The most commonly investigated variants within *RNASEL*, R462Q and D541E, have been found to be significantly associated with CaP in several studies [111,112,113], while other reports show a lack of association for both SNPs and CaP [114,115]. A meta-analysis of 10 independent *RNASEL* genotyping studies for the variants E265X, R462Q, and D541E found an association of the D541E variant with a less than two-fold increased risk of CaP in Caucasians, irrespective of family history [113]. Several independent analyses of AA CaP families were able to confirm the contribution of the HPC1 locus [116,117] to increased CaP susceptibility in AA men. Results from a study of R462Q and D541E variants in a cohort of non-Hispanic Caucasian, Hispanic Caucasian, and AA CaP cases and controls supported the role of *RNASEL* as a predisposition gene for CaP and found significant association between the 462Q variant and CaP risk in AA and Hispanic Caucasians [112].

In addition to its linkage to hereditary CaP, the macrophage receptor scavenger 1 (*MSR1*) gene is located at a locus (8p22) that is often deleted in prostate tumors [118,119]. *MSR1* mutations have been associated with CaP risk in both hereditary and sporadic CaP among Caucasian and AA men [98]. A meta-analysis of eight studies that evaluated common *MSR1* mutations and sequence variants, stratified by race, and sporadic or hereditary cancer concluded that the *MSR1* gene does not independently confer a major risk to CaP but may confer a moderate risk to CaP, especially in black men [120]. An association study of tagged SNPs in *ELAC2*, *RNASEL* and *MSR1* for suggests that interactions among these genes contribute increased CaP risk consistent with a polygenic model of CaP susceptibility [121].

### 4.2. HOXB13

The *HOXB13* gene codes for a homeobox related transcription factor that regulates gene expression cascades regulating critical cell growth and differentiation stages during prostate development [122]. HOXB13 protein has been shown to be critical for cellular response through its interaction with Androgen Receptor (AR) and FOXA1, and transcriptional inhibition of AR regulated genes [123,124]. The *HOXB13* G84E (rs138213197) variant appears to be the first major germline mutation associated with high risk of the hereditary prostate cancer (HPC). Initially mapped to the 17q21–22 regions by linkage analysis, the rare but recurrent mutation was identified in 18 individuals from four different families after sequencing 200 genes [29]. The mutation rate was approximately twenty-fold higher in unrelated CaP cases of European descent (1.4% or 72 in 5083) compared to control subjects (0.07% or 1 in 1401). The mutation confers an odds ratio of 5.1 for the development of CaP among men with positive family history and early onset compared to 1.7 among men with no family history and late onset [29]. In another genotyping study, the discovery of at least one *HOXB13* G84E mutation carrier in 112 families out of 2443 CaP families (4.6%), primarily of European descent, also supported the association of this mutation with CaP [125]. Other studies further confirmed that the *HOXB13* G84E mutation substantially increases risk of early onset familial CaP in CA men [102,126,127,128] (Figure 2). The G84E mutation was found to be most prevalent in families from the Nordic countries of Finland (22.4%) and Sweden (8.2%) [129]. Different *HOXB13* mutations have also been detected in CaP cases in other racial or ethnic groups, including in African (G216C and R229G) [29] and Asian (G135E) populations [130], but the frequency and impact of these mutations on the risk of CaP remains to be confirmed in studies involving larger cohorts.

### 4.3. BRCA1 and BRCA2

*BRCA1/2* are tumor suppressor genes (TSG) and the germline mutations of either results in an increased risk of ovarian cancer and early onset breast cancer associated with hereditary breast and ovarian cancer (HBOC) syndrome [131]. The proteins encoded by *BRCA1* and *BRCA2* protect the genome by using the homologous recombination (HR) pathway to carry out high-fidelity replication associated double-strand break (DSB) repair, relying on the undamaged sister chromatid as a template DNA [132]. BRCA1 protein has a broad range of functions that include recruiting effectors to DSB sites, mediating the end resection of DSBs, activating the G1/S, S-phase, and G2/M checkpoints, and mediating HR, Non-homologous end joining (NHEJ), as well as single-strand annealing (SSA) repair pathways [132,133]. BRCA2 functions primarily in facilitating HR by recruiting RAD51 to sites of DSBs and in repairing DSBs by HR60 [134]. Rare variants in *BRCA2* and *BRCA1* were implicated to give rise to moderately elevated risk to CaP [59,73,135]. Deleterious mutations in *BRCA1* and *BRCA2* have also been shown to increase the risk of CaP [136].

*BRCA1* mutation carriers younger than 65 years have been shown to have a two-fold increased relative risk of CaP but no evidence of an elevated risk in men aged 65 or older [137,138]. A substitution in *BRCA1* at Q356R was shown to be preferentially transmitted to affected men from 323 non-Hispanic white families with familial and early-onset CaP at an estimated odds ratio of 2.25 [139]. Several studies have evaluated the contribution of *BRCA1* (185delAG and 5382insC) and *BRCA2* (6174delT) founder mutations to risk of CaP among Ashkenazi Jewish men [140,141,142]. Although deleterious mutations for both genes were more prevalent in CaP cases compared to controls in this population, *BRCA2* mutations were found to confer at least a 3-fold elevated risk of high grade CaP, while evidence for the involvement of *BRCA1* in increased risk of CaP was modest [142]. Nevertheless, an evaluation for carriers of *BRCA1* mutations in Hispanic, AA, and Asian American breast cancer patients compared with non-Hispanic white patients, with and without Ashkenazi Jewish ancestry, indicated a prevalence of pathogenic *BRCA1* mutations in minority racial populations [143]. 

**Figure 3:**
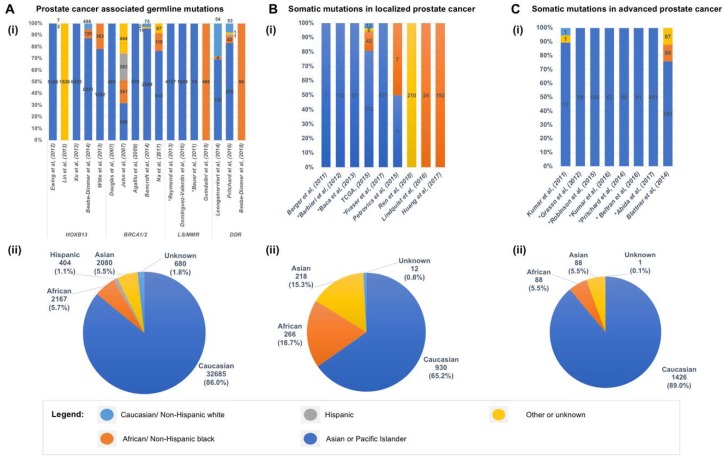
Racial and ethnic disparity in genomic studies of prostate cancer. The distribution of race and ethnicity of cohorts analyzed for germline mutations (**A**), somatic mutations in localized prostate cancers (**B**), and somatic mutations in advanced or metastatic castrate resistant prostate cancers (**C**), in each study reviewed (i), and when grouped together (ii). * In studies which the race or ethnicity of cohort were not specified, the cohort were assumed to be largely Caucasian based on the population of the locale where the study was conducted.

Germline mutations in *BRCA2*, which have been shown to be associated with early onset and poor prognosis of CaP, may account for about 5% of CaP in familial clusters [144,145,146]. An evaluation of 12 CaP cases from 16 Icelandic families who were carriers of a *BRCA2* founder mutations, detected the mutation in 8 patients (66.7%), all of whom developed advanced disease and succumbed to CaP [144]. An analysis of the risk of *BRCA2* mutations for development of other cancers in 173 breast-ovarian cancer families found a five-fold increased relative risk of CaP, which increased to seven-fold among men below the age of 65 among *BRCA2* mutation carriers compared with non-carriers [138]. A study on the benefit of targeted screening of *BRCA1/2* carriers for earlier detection of CaP, men 40 to 69 of age with germline *BRCA1/2* mutations and a non-carrier control group were biopsied if their PSA exceeds >3 ng/mL. Initial results indicated higher positive predictive value (PPV) for PSA triggered biopsy in *BRCA2* carriers (PPV 48%) compared to controls (PPV 33%), and two-fold higher incidence of CaP (3.3%) in carriers compared to controls (1.6%) [147]. Men with a *BRCA2* mutation were found to have a poorer survival rate (61.8%) than those without a *BRCA2* mutation (94.3%) over a 12-year period [148]. A meta-analysis of the association between *BRCA* mutations and CaP risk and prognosis confirmed that *BRCA1* and *BRCA2* mutations confer up to 4.5-fold and 8.3-fold increased risk of CaP, respectively. *BRCA2* mutations were also found to be associated with an increased risk of high-grade disease, progression to metastatic castrate resistant prostate cancer (mCRPC), and 5-year cancer-specific survival rates of 50% to 60% [149]. The results of these studies showed that a subset of men with early-onset CaP will carry *BRCA1* or *BRCA2* mutations, and that germline mutations of *BRCA2* have a greater contribution to an increased risk of CaP compared to those in *BRCA1*. The small numbers of mutation carriers available has hampered the efforts to determine the frequencies of *BRCA1* and *BRCA2* germline mutations in AA and other racial groups [103] (Figure 3A,B). In view of increased sensitivity of tumors harboring *BRCA1/2* mutations to Poly (ADP-ribose) polymerase (PARP) inhibitors, which can extend the overall survival in mCRPC patients with *BRCA1/2* mutations, the early identification of *BRCA1/2* mutation carriers and stratification for targeted therapy could improve survival in localized CaP patients. Thus, an awareness of existing family history of cancer in men who present with early-onset CaP could identify this risk and help to avoid harmful clinical consequences. 

### 4.4. DNA Mismatch Repair (MMR) Genes

Evidence from recent studies suggests that men who are carriers of germline mutations of DNA mismatch repair (MMR) genes have an increased risk of CaP. Mutations in MMR genes, including *MSH2*, *MLH1*, *PMS1*, *PMS2* or *MSH6*, are associated with Lynch Syndrome (LS), a highly penetrant autosomal dominant cancer predisposition syndrome characterized by hereditary nonpolyposis colorectal cancer and other neoplasms. Inactivation of MMR proteins, which work together to repair base-base mismatches and insertion/deletion mispairs generated during DNA replication and recombination, result in a high rate of microsatellite instability (MSI) in their tumors [99]. In a study conducted in Norway, carriers or obligate carriers of MMR gene mutations developed CaP earlier and at a higher frequency than expected to occur by chance in the population [150]. Another study of 764 MMR gene mutation carriers previously diagnosed with colorectal cancer, estimated a 3% higher risk of CaP compared to the general population, demonstrating that carriers of MMR gene mutations with colorectal cancer are at increased risk of CaP [151]. Results from a separate study showed a two-fold higher cumulative lifetime risk of CaP in men with LS compared to the general population [104]. An evaluation of CaP in 16 mutation carriers and in 12 first-degree relatives from 288 Danish LS families found that tumors with mutations in *MSH2*, *MLH1*, and *MSH6* genes had higher Gleason grades, displayed MSI-high phenotype and loss of the respective MMR proteins in a subset of the tumors, further demonstrating the link between LS and CaP [105]. The detection of MSI in prostate tumors of men who were MMR mutation carriers, but not in others with hereditary or sporadic CaP, suggests a causative role in defective DNA mismatch repair [152]. 

Due to the low frequency of mutations in *MMR* genes, studies that include a diverse racial or ethnic cohort are limited, and the frequencies of MMR genes in minority populations and their association with increased CaP risk remains to be understood [106,153] (Figure 3A,B). A characterization of the mutation spectrum among 51 AA families from the Colon Cancer Family Registry with LS reported the detection of several recurrent and novel mutations in MMR genes [106]. They noted a predominance of mutations in *MLH1* (61%) followed by *MSH2* (21%) among AA cases, whereas among Caucasians with LS, the reported predominant phenotype is *MSH2* followed by *MLH1*. Remarkably, the frequency of the *MLH1* mutation among Caucasians from the same registry was 29.5%. Compared with the AA SEER population-based data, AA men who are MMR gene mutation carriers, and *MLH1* and *MSH2* mutation carriers presented significantly increased risks for CaP [106]. 

### 4.5. Germline Alteration in DNA Damage Repair Pathways

A common thread that links the hereditary breast-ovarian cancer syndrome and Lynch Syndrome associated hereditary nonpolyposis colorectal cancer is mutations of genes in the DNA Damage Repair (DDR) pathway. In addition to *BRCA1* and *BRCA2*, germline mutations in several other DDR genes have been reported to potentially confer greater risks of CaP. The *CHEK2* TSG, which encodes the Checkpoint Kinase 2 (CHK2) protein, is activated by DNA damage to interact with p53, leading to cell cycle arrest, allowing the cell to repair damaged DNA or undergo apoptosis. Missense variants of *CHEK2* have been found to occur between 3% to 10% of CaP cases and are associated with an increased risk of CaP [154,155]. A genotyping study of 637 CaP patients and 445 controls identified the *ATM* 3161G (P1054R) variant allele to be significantly associated with an increased risk of developing CaP (odds ratio = 2.13) [156]. ATM is recruited to DSB sites where it activates other sensor proteins (MDC1 and γH2A.X), as well as CHK2 and p53, which results in cell-cycle arrest to allow the cell to repair the DSB instead of undergoing apoptosis [157]. In a targeted sequencing of 22 TSGs in germline DNA from 191 men with hereditary CaP, deleterious mutation in one of these genes were found in 14 men (7.3%), who were also more likely to have clinically advanced disease. The most commonly mutated genes of the 22 tested were *BRCA2*, *ATM*, *CHEK2*, and *BRIP1/FANCJ* [100]. In a recent integrated analysis of 150 mCRPC cases for pathogenic somatic and germline genomic alterations, which include alterations in *BRCA2*, *BRCA1* and *ATM*, were detected in 8% of the cases [158]. A larger study by Pritchard et al. [30] of 692 men with mCRPC identified a significantly higher incidence (11.8%) of germline mutations of DNA-repair genes among men with mCRPC than the incidence (4.6%) among men with localized CaP. Deleterious germline mutations of DNA-repair genes were detected in 16 genes, including *BRCA2* (5.3%), *ATM* (1.6%), *CHEK2* (1.9%), *BRCA1* (0.9%), *RAD51D* (0.4%), and *PALB2* (0.4%). Increasing evidence has demonstrated that these germline DNA-repair gene mutation carriers are at increased likelihood of experiencing advanced disease, metastatic spread, and poorer survival outcome. 

Studies of the contribution of rare genetic variants of DDR genes to CaP in AA men and other minority populations have been hampered by low participation in large genetic studies, particularly those focused on early-onset and familial disease [30,100] (Figure 3A,B). Beebe-Dimmer and colleagues performed targeted exome sequencing of 160 genes in a cohort of 96 AA men with early-onset CaP (≤55 years at diagnosis) to characterize rare germline mutations in young AA men diagnosed with clinically significant CaP. They identified protein-truncating mutations in *BRCA2* and *BRIP1/FANCJ* in three men with early onset CaP, as well as rare, missense variants in *BRCA1*, *BRCA2*, *PMS2*, and *ATM* that were likely to be pathogenic [101].

## 5. Somatic Mutations in the Genome of Localized Primary Prostate Cancer

Before the advent of Next-Gen sequencing (NGS) technologies, alterations of somatic mutation patterns and signaling pathways in primary and metastatic tumors were detected mainly by gene expression and copy number profiling studies using array based technologies followed by targeted sequencing of a limited number of samples [159,160,161,162]. Classical and molecular cytogenetic techniques, including fluorescent in-situ hybridization (FISH), and comparative genomic hybridization (CGH), were also used to detect gene deletions and duplications, such as the deletions of *NKX3-1* at 8p21 and *PTEN* at 10q23, and amplifications of *c-MYC* at 8q24 and *AR* at Xq12 [163,164]. The higher frequencies of *BRAF* and *RAF1* copy number gain in CaP among Chinese men than in CA men were detected by using FISH [165]. Further advances in gene expression profiling and bioinformatics have revealed the overexpression of ERG in prostate tumors that led to the discovery of *TMPRSS2-ERG* fusion [166,167]. The evolution in NGS and computing technologies, together with collaborative efforts of cancer researchers have accelerated the profiling of the genomic landscape of localized and metastatic prostate tumors [168,169]. These efforts have confirmed the mutation of frequently altered driver genes in CaP, including *TMPRSS2-ERG* gene fusion and *PTEN* deletion.

The initial genomic studies of CaP using NGS technologies were performed largely on tumor specimens from men of Caucasian or European ancestry (Figure 3C,D). The first WGS on primary prostate tumors of seven CA men by Berger et al. [170] identified mutations in *Speckle-Type POZ Protein* (*SPOP*) gene, subunit of a Cullin-based E3 ubiquitin ligase, and in genes coding for chromatin modifiers (*CHD1*, *CHD5*, and *HDAC9*), and heat shock stress response chaperone complex (*HSPA2*, *HSPA5*, and *HSP90AB1*). The study uncovered, in addition to *PTEN*, novel deletions in *CADM2* and *MAGI2*. The use of NGS in this study enabled the observation of balanced chromosomal rearrangements, which were not detected using earlier methods. A subsequent WES on 112 localized prostate tumors from primarily Caucasian patients identified new recurrent mutations in *MED12* and *FOXA1* [171]. The authors highlighted *SPOP* as the most prevalent gene mutation (13%), which was mutually exclusive of *ETS* family gene rearrangements and was associated with distinct mutation profiles that defined a new molecular subtype of CaP. The WGS analysis of a subset of this cohort, consisting of 57 genomes of prostate tumors, identified *TMPRSS2-ERG* fusions, and recurrent deletion or rearrangement of cancer driver genes *PTEN*, *NKX3-1*, *CDKN1B*, *TP53*, and *RB1* [172]. The term “chromoplexy” was introduced to describe a pattern of highly interdependent DNA translocations and deletions that generated oncogenic fusions and disrupted multiple TSGs over relatively few events, which became the basis of the proposed model of punctuated cancer evolution [172].

A coordinated effort by The Cancer Genome Atlas (TCGA) researchers analyzed 333 primary tumors by WES and an additional 119 cases by WGS [173]. Their efforts resulted in the grouping of 74% of the tumors into one of seven molecular subtypes based on distinct oncogenic drivers, defined by gene fusions involving genes of the *ETS* transcription factor family (*ERG*, *ETV1*, *ETV4*, or *FLI1)*, or mutations in *SPOP*, *FOXA1* or *IDH1*. These subgroups have unique Androgen Receptor (AR) signaling response, genome-wide DNA hyper-methylation, and miRNA expression profiles [173]. Mutations or deletions in DNA repair genes, such as *BRCA2*, *BRCA1*, *CDK12*, *ATM*, *FANCD2*, or *RAD51C* affected about 20% of cases. Approximately 25% of the tumors had alterations in the *PI3K* or *MAPK* signaling pathways, with frequent loss of *PTEN* (17%), or activation of *PIK3CA*, *PIK3CB*, *AKT1*, and *MTOR*. In addition to identifying the major subtypes among primary prostate cancers, results from this study revealed substantial molecular heterogeneity and underscored potentially actionable molecular defects. Although this study included prostate tumors from 43 (13%) AA men, the number remains insufficient for the findings to be extrapolated to AA or other minority patient groups. 

**Figure 4:**
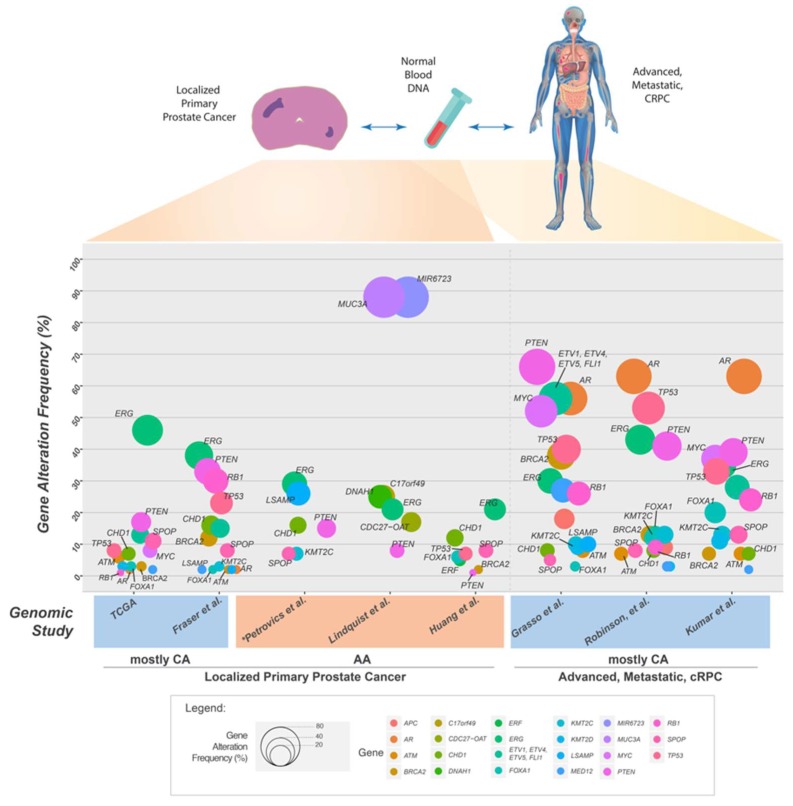
Frequency of somatic gene alteration events reported in selected genomic studies of localized primary prostate cancer and in advanced, metastatic, and/or castrate resistant prostate cancer. Size of circle is proportional to the frequency of gene alteration events (point mutations, deletions, amplifications or gene fusions). * Only gene alteration events in African American cases are shown.

The largest study of primary prostate cancers to date, which focused on localized and non-indolent CaP genome of Gleason Sum less than 7, was carried out by the Canadian CaP Genome Network (CPC-GENE), in partnership with the International Cancer Genome Consortium (ICGC) [174]. The researchers analyzed a total of 200 cases by WGS, and an additional 277 cases by WES in order to achieve near-saturation identification of genomic alterations. A profiling of CNAs in 284 cases found recurrent allelic gains of *MYC* and deletions of *PTEN*, *TP53*, and *NKX3-1* that corroborated with earlier reports. Considerably fewer clinically actionable single nucleotide variants (SNVs) were observed in the primary tumors analyzed than in metastatic disease. Only six genes had coding SNV mutations in more than 2% of tumors: *SPOP* (8.0%), *TTN* (4.4%), *TP53* (3.4%), *MUC16* (2.5%), *MED12* (2.3%), and *FOXA1* (2.3%) (Figure 4). Large-scale genomic rearrangements, including *TMPRSS2*-*ERG* fusion were found in 38% of cases. “Kataegis” and “chromothripsis”, defined by DNA single-strand breaks and double-strand breaks, respectively, were frequent and correlated with specific genomic profiles [175,176]. SNV mutations in *ATM* were found to be predictive of patient outcome: all patients suffered relapse as defined by biochemical recurrence. Although the findings were based on a cohort of largely European descent, this study highlights the biological difference of localized prostate cancers from advanced mCRPC. The scarcity of recurrent SNV driver aberrations in localized disease led the authors to suggest widespread genotoxic chemotherapy for genetically unstable localized tumors requiring intensified therapy.

## 6. Somatic Mutations in the Genome of Prostate Cancers from African American Men

Recent discoveries that highlight the higher frequencies of *TMPRSS2-ERG* gene fusion and *PTEN* deletion in prostate tumors of CA men compared to AA men increasingly support the idea that CaP of AA and CA men have distinct somatic gene alterations [166,177,178,179]. Petrovics et al. [180] were the first to compare the genomic profile of localized prostate tumors between seven AA and seven CA men by WGS. Somatic SNVs were detected in coding sequences of *SPOP*, *MED12*, *TP53*, *KMT2C*, *ATM*, *CTNNB1*, and *PIK3CB*, genes previously identified to be recurrently mutated in prostate or other cancers. In addition to confirming the presence of recurrent CaP genomic alterations such as *TMPRSS2-ERG fusion*, *PTEN* and *CHD1* deletions, a novel recurrent deletion of *LSAMP* gene on chromosome 3q13.31 was found to more prevalent in prostate tumors from AA men compared to CA men (26% vs. 7%). *LSAMP* deletion was associated with rapid disease progression, defined by earlier incidence of biochemical recurrence. Deletions of *LSAMP* in other cancers have been associated with aggressive disease [181,182]. A recent study of CaP genome in a Chinese cohort detected *LSAMP* alterations at a frequency (13 of 65 cases, 20%) comparable to that detected in this AA cohort [183]. In contrast, *PTEN* deletion was confirmed to be more prevalent among CA (63%) than AA (15%) men by FISH analysis on tissue microarray from an independent cohort. *PTEN* deletion, together with the activation of *ERG* oncogene via gene fusions, makes up the early tumorigenic driver genes that occur at higher frequencies in CA patients compared to AA men [177,178,179]. The distinct prevalence of recurrent genomic alterations of *PTEN*, *ERG*, and *LSAMP* between AA and CA CaP were further validated by cumulative evaluation of data from 435 cases using FISH analyses of tissue microarrays, and SNP array data from TCGA. 

Frequent deletions of *CHD1* in CaP define a subtype of CaP characterized not only by association with *SPOP* mutations and *ETS* gene family fusion-negative status [184,185] but also increased genomic instability [186] that contribute towards development of aggressive CaP [187]. The chromatin remodeling function of CHD1 facilitates the recruitment of HR proteins to DSB sites, specifically C-terminal-binding protein interacting protein (CtIP) and 53BP1, and subsequent end resection during DNA DSB repair [188,189]. Consequently, the loss of *CHD1* leads to decreased error-free HR repair, and resultant increased error-prone NHEJ repair of DSB. These events sensitize cells to PARP inhibitors, which has potential therapeutic relevance.

Lindquist et al. analyzed the genomes of aggressive CaP from 24 AA patients with Gleason grades ≥7 and pathologic stage ≥T2b [190]. The lower prevalence of *TMPRSS2-ERG* gene fusions (21%), and *PTEN* deletions (8%), as well as patterns of Copy Number Alterations (CNAs) exemplified by losses primarily in 8p and gains in 8q, corroborated with previous CaP studies [177,178,179,191]. This study highlighted a novel gene fusion involving *CDC27*-*OAT*, present in 4 of 24 patients (17%). CNAs involving the amplification of *MIR6723*, *PCBD2*, and *TXNDC15*, and the loss of *EBF2* were also detected. 

A recent genomic analysis of localized primary prostate tumors on a discovery set 102 AA cases by WES, followed by targeted sequencing on an extension set of 90 cases identified novel recurrent loss-of-function mutations in *ERF* in 5% of AA cases [192] (Figure 3C,D). Deletions of *ERF*, which codes for an *ETS* transcriptional repressor, were detected in 3% of primary prostate cancers, while 3% to 5% of lethal castration resistant prostate adenocarcinomas (CRPCs) harbor either mutations or deletions in *ERF*. Significant levels of somatic mutations were also detected in *SPOP* and *FOXA1.* Consistent with published reports, deletion of *PTEN* or mutation of *PIK3CA* that disrupts the PI3K signaling pathway prove to be infrequent in the primary AA cohort analyzed: the deletion frequency of *PTEN* (6%) in the AA discovery cohort of was less prevalent than in the TCGA cohort (32%); no mutation in *PIK3CA* was detected, compared to approximately 3% mutation frequency in TCGA primary prostate cancers. 

## 7. Genomic Landscape of Advanced and Metastatic Castrate Resistant Prostate Cancer (mCRPC)

Kumar et al. [193] were among the first to apply NGS to the genomic analysis mCPRCs by evaluating 23 CaP xenografts derived from 16 different advanced metastatic tumors and high-grade primary carcinomas of CA patients using WES (Figure 3E,F). In addition to recurrent non-synonymous somatic and germline mutations in *TP53*, *DLK2*, *GPC6*, and *SDF4* genes, they observed mutations in the WNT pathway and cases with excessive point mutations or “hypermutated” phenotype. Grasso et al. further delineated the mutational landscape driving the progression of CaP to lethal mCRPC [185], by carrying out WES on 50 lethal, heavily-pretreated mCRPCs. Their analyses confirmed the monoclonal origin of lethal CRPC and noted low overall mutation rates (2.00 per megabase) in heavily treated mCRPCs. This study highlighted deletions in *CHD1* (defined a subset of tumors mutually exclusive of *ETS* family gene fusions), and recurrent mutations in multiple chromatin- and histone-modifying genes, including *KMT2D.* Mutations were also detected in a number of genes coding for proteins that physically interact with the AR, such as *ERG*, *FOXA1*, *KMT2D*, *KDM6A*, and *ASXL1*, which impacts AR-mediated signaling function, highlighting the disruption of novel AR signaling mechanisms in mCRPC tumors (Figure 4). 

A larger effort to evaluate the genomic landscape of mCRPC multi-institutional by the Stand Up To Cancer (SU2C)-Prostate Cancer Foundation (PCF) team was carried out by performing whole-exome and transcriptome sequencing of 150 biopsies from mCRPC affected individuals [158]. They confirmed the higher prevalence of mutations in *AR* (63%), *ETS* family (57%), *TP53* (53%), and *PTEN*-*PI3K* pathway genes (49%) compared to localized CaP (Figure 4). The inactivation of critical DNA repair genes was detected in 23% of mCRPC cases. Genes critical for HR mediated repair, *BRCA2*, *ATM*, and *BRCA1*, comprised 19% of the mCRPC cases. Three out of four mCRPC tumors showed a hypermutation phenotype and harbored mutations in the MMR pathway genes *MLH1* or *MSH2*, in agreement with a recent report identifying *MSH2* and *MSH6* alterations in hypermutated CaP [194].

In an effort to evaluate intra-patient tumor heterogeneity, Kumar et al. compared the genomic diversity of 176 primary or metastatic CaP tumors within and between individuals from 63 men previously treated with androgen-deprivation therapy, by WES, array CGH, and transcriptome profiling [195]. Notably, mutation or amplification of the *AR* gene, which is extremely rare in untreated primary CaP, was detected in 63% of mCRPC (Figure 4), comparable to the frequency reported by Robinson et al. [158]. Despite extensive prior treatment to suppress AR function, 88% of men had tumors with robust AR activity and AR activity was inversely associated with cell proliferation [195]. Men with somatic aberrations in DNA-repair genes, such as Fanconi anemia (FA)-complex genes or *ATM* were found to respond over a longer period to carboplatin treatment. In contrast to the increased heterogeneity in primary tumors between individuals, there was limited diversity among metastases within an individual. These findings suggest that major oncogenic driver alterations in metastatic tumors within an individual could be identified by evaluating a single metastasis, which could be used to guide treatment options based on the predicted molecular vulnerabilities. 

Progress in NGS has facilitated the comparison of localized and metastatic CaP genomes that enhanced our appreciation of the levels of CaP heterogeneity. Although metastatic tumors display a significantly higher rate of CNAs and mutation frequencies [158,185,195], the subtype distribution remained fairly similar, except for the scarcity of *IDH1* mutation in metastatic tumors [158,173]. In primary tumors, increased AR-driven transcription activity was found to be associated with *SPOP* or *FOXA1* mutation subtypes [173]. Interestingly, AR signaling was more frequently altered in the metastatic tumors, by amplification or mutation of AR, events that are usually absent in primary tumors, indicating that most mCRPC tumors have active AR signaling pathway. Treatment of patients with potent AR-pathway antagonists, such as abiraterone or enzalutamide, could induce diverse resistance mechanisms, including *AR* amplification, *AR* mutation, and expression of *AR* splice variants, or select for distinct phenotypes that may become insensitive to AR signaling [196,197]. These results suggest a need to further increase our understanding of the genetic contributions to aggressive or lethal CaP.

To better understand the molecular basis of a subset of tumors that are insensitive to androgen and display neuroendocrine features, Beltran et al. [198] performed WES on 114 metastatic tumor specimens from 51 men with castration resistant prostate adenocarcinoma (CRPC), and on 30 with castration resistant neuroendocrine prostate cancer (CR-NEPC). The significant overlap between CRPC and CR-NEPC tumor genomes observed fits a model most consistent with divergent clonal evolution. It was proposed that CR-NEPC is most likely to have adapted from one or more subclonal populations of CRPC cells with wild-type *AR* under selective pressure and subsequently acquired new genomic and epigenomic drivers associated with decreased AR signaling and epithelial plasticity [198]. In an independent study, targeted deep sequencing was performed on 504 tumors, ranging from localized, metastatic non-castrate, and mCRPC, from 451 patients. Somatic alterations in *TP53* and *BRCA2* were identified as early tumorigenesis events, while alterations in *APC* and *ATM* were enriched in metastatic and CRPC tumors, respectively [199]. About 27% of patients were found to harbor a germline or somatic mutation of a DDR pathway gene. Additional potentially actionable mutations were identified in PI3K and MAP kinase pathways.

*SPOP* mutations, which are more frequent in localized primary tumors (13%) compared to metastatic tumors (8%), identify a distinct molecular subclass of CaP that is almost mutually exclusive of ETS family rearrangements. Besides elevated levels of DNA methylation, and *SPINK1* mRNA overexpression, cases with *SPOP* mutations are associated with higher rates of CHD1 deletions (occurring in 58% of cases) [158,171,173]. A targeted evaluation of *SPOP* mutations of CaP tissue specimens derived from radical prostatectomy, transurethral resection of the prostate, or metastatic biopsies from a multi-ethnic cohort of 720 CA, AA, and Asian patients detected *SPOP* mutations occurring at a frequency of 8.1% (4.6% to 14.4%), in agreement with earlier reports [184]. However, no significant association between *SPOP* mutations with ethnicity, clinical, or pathologic parameters was observed. Interestingly, Romanel et al. identified a non-coding polymorphic allele at 7p14.3, associated with *SPOP* mutations through a hormone dependent DNA damage response [200]. This genetic predisposition may positively select for *SPOP* mutations in response to DNA damage.

## 8. Impact of Prostate Cancer Genomics on the Prognosis, Treatment, and Ethnic Disparity of Prostate Cancer

The availability of high-fidelity genomic sequence data from NGS has not only revealed the differences between intra-patient and inter-patient tumor heterogeneity [195] but also the differences between intra-focal and inter-focal tumor heterogeneity within an individual [201]. The identification of deleterious germline and somatic mutations in the CaP genome has provided a basis for stratifying of tumors into molecular subtypes with distinct genomic alteration profiles [31,173]. This practice could provide the framework for improving the prognosis of disease and developing predictive biomarkers for personalized treatments. Genomic alterations of distinct driver genes may reveal critical signaling pathways and tumor-specific dependencies that could be targeted with treatments currently in development for other cancers. 

The DDR pathway provides an example of a clinically actionable pathway that is responsive to targeted treatments [103,202,203]. The enrichment of DDR gene defects in mCRPC tumors suggests a possible role for genomic instability in promoting carcinogenesis, disease progression, and development of a more aggressive phenotype. Carriers of germline *BRCA2* mutation are more likely to present with advanced CaP with higher Gleason score, and exhibit poorer survival than non-carriers [103,144,148,149]. Somatic mutations of DDR genes affected approximately 20% of localized primary CaP and mCRPC [158,173]. Germline DNA alterations to DDR genes were detected in approximately 10% of mCRPC tumors [30,158]. Initially developed as an anticancer agent for tumors with impaired homologous recombination DNA repair, PARP inhibitors have been shown to induce significant tumor responses in cancer patients carrying germline *BRCA1*/*2* mutations [204]. Metastatic CRPC tumors harboring germline or somatic defects in DDR genes have been reported to be susceptible to PARP inhibitors through a synthetic lethal interaction [204,205] and have also shown hypersensitivity to platinum-based chemotherapy resulting in exceptional responses [195,206,207]. Defective DNA repair genes may confer tumor-specific vulnerability to immune checkpoint inhibitors, due to a higher neo-antigen burden resulting from genomic instability in these patients’ tumors [208,209]. 

Mutations that disrupt the RAS-Phosphatidylinositol 3-kinase (PI3K)-and mitogen-activated protein kinase (MAPK) pathways mostly through the deletion of *PTEN* or rare mutations in other pathway members, including *PIK3CA*, *PIK3CB*, *AKT*, and *MTOR*, also provide targets for potential treatment. Gene alterations of this signaling pathway were found in approximately 25% of primary tumors and in about 50% of mCRPC tumors but were less frequent in primary CaP of AA men [158,173,190]. Currently, several small molecule inhibitors targeting the PI3K signaling pathway, such as the dual PI3 Kinase/mTOR inhibitor LY3023414, and the PI3K-β inhibitor (GSK2636771) in combination with enzalutamide, are being evaluated in patients harboring tumors with *PTEN* loss [210,211]. 

The hypothesis that the presence of a molecular marker predicts response to a targeted therapy regardless of tumor histology has become the basis for clinical trials referred to as “basket trials” [212]. The NCI-MATCH (Molecular Analysis for Therapy Choice) currently in progress, is an example of a basket trial that plans to match patients harboring any solid tumor or lymphoma with alterations in at least one of a panel of cancer driver genes with a targeted drug, independent of tumor histology [213]. Such trials aim to establish proof of principle for developing a precision medicine approach to treat clinical cancer by identifying novel targets that are responsive to treatment. The recent US Food and Drug Administration approval of immune checkpoint inhibitors for microsatellite instability-high (MSI-hi) and MMR-deficient cancers has direct implications for CaP with similar genetic profiles [208,209]. These developments suggest that it may soon become feasible to routinely match the genomic information of the patient and their tumors to the most efficacious treatment available. 

The feasibility of targeted therapies for CaP is likely to create a demand for routine genetic profiling of tumor and germline DNA by next-generation sequencing assays, particularly in the management of advanced or mCRPC [205]. More patients with mCRPC are likely to have their germline or tumor DNA sequenced to find actionable mutations that can guide individualized treatment options. These decisions could lead to the discovery of germline mutations that may have wider implications on immediate family members, who in turn may require genetic counseling [199,205]. Based on the need to inform precision medicine options and the increasing evidence for a hereditary basis for CaP, the Philadelphia Prostate Cancer Consensus 2017 was convened to establish guidelines for genetic testing for inherited CaP risk, genetic counseling, and management on the basis of genetic findings [80]. The panel strongly agreed on the need for engagement of patients in shared decision making for genetic testing for CaP. The panel achieved a strong consensus for the targeted testing of specific genes in all men with CaP, who are from families with higher risks conferred by hereditary prostate cancer (HPC), hereditary breast and ovarian cancer (HBOC), or Lynch Syndrome (LS), which involves the testing of *HOXB13*, *BRCA1/2*, and DNA mismatch repair genes, respectively. There was moderate agreement to test *ATM*, but a strong consensus to factor in *BRCA2* for the screening of CaP to inform prognosis and targeted therapy. While acknowledging the urgent need to identify distinct germline mutations that confer risk of hereditary CaP among AA men, there was strong consensus that until such genetic data are available, AA men should follow the same criteria as men in other race groups. This consensus convention is a milestone event marking the threshold of a new era for the multi-gene testing for CaP.

## 9. Addressing the Under-Representation of Minority Populations in CaP Genomic Studies

This review has discussed the progress from early identification of germline variants associated with increased susceptibility to CaP to recent profiling of germline and somatic alterations leading to the molecular subtyping of tumors for personalized treatments. The recent advances in genomic sequencing of cancer types, including CaP, has revealed a deficit in the participation of racial and ethnic minorities. A survey of the over five thousand samples that were sequenced in ten tumor types within The Cancer Genome Atlas (TCGA) initiative, only 12% were from Blacks, 3% were Asian, 3% were Hispanic and less than 0.5% were from Native Hawaiian, Pacific Islander or Native Americans compared to 77% from Whites [18]. The breakdown of racial or ethnic representation of studies on hereditary cancer syndromes and in the mapping of the genomic landscape of both localized and metastatic prostate tumors discussed in this review further underscore this fact. The inclusion of AA men and other minority populations can identify population specific variants to help us better understand their contribution to overall cancer burden [214]. Large-scale collaborative genomic studies of CaP that address the under-representation of AA and minority populations are essential for the discovery and validation of distinct cancer driver gene alterations across populations of different ancestries. To detect such unique mutations in a subpopulation of patients we have to be able to discern a signal above the noise–the mutation rate of the gene of interest above the background mutation frequency. Thus, large sample sizes are required to establish the power to detect mutations confidently above the background rate [215]. The underlying diversity in allelic architecture or differential linkage dis-equilibrium across population raises doubt whether genetic discoveries derived from cohorts underrepresented by minority groups could be generalized across populations and that the translation of these findings into pan-ethnic clinical and public health interventions may worsen existing health disparities [216]. Recent reports of a higher prevalence of novel *LSAMP* deletion [180], *CDC27*-*OAT* gene fusion [190], and deleterious *ERF* mutations [192] in CaP tumors of AA men, and increased *PCDH9* deletion and *PLXNA1* amplification in CaP tumors of Chinese men [183], highlight the presence of distinct CaP genomic profiles of men from different racial or ethnic populations. Whether these genomic aberrations are prevalent in AA or other ethnic populations and could become actionable targets for personalized treatments would require further validation in large-scale studies. 

In the US, concerns over the lack of diversity in genetic research has led to the mandated inclusion of minority groups under the National Institutes of Health (NIH) Policy and Guidelines on Inclusion of Women and Minority as Subjects in Clinical Research [217]. Scientist are required to use the race categories defined by the U.S. Office of Management and Budget (OMB): American Indian or Alaska Native, Asian, Black or African American, Hawaiian or Pacific Islander, and White [218]. Hispanic or Latino identity are separately classified under ethnicity. These categories are used to collect, present and compare data on race and ethnicity amongst federal databases, including the census and national health databases. However, these are oversimplified social-political constructs that neither accurately define the anthropological or the genetic diversity of human populations. For example, a Pew Research Center survey estimated that 6.9% of American adults are multiracial based on races reported for themselves, their parents and their grandparents, and if the races of great-grandparents and earlier ancestors were taken into account, that estimate would have increased to 13.1% [219]. In addition to homogenous races of people that arise from geographic or cultural isolation over many generations, population processes that include migration, colonization, conquest, slavery, socially enforced endogamy and natural selection, have contributed to the patterns of human genetic variation that exists today [220,221]. Although the term “ethnicity” was often used interchangeably with race, it emphasizes the cultural, socioeconomic, religious status of human groups rather than their genetic heritage, and it may change with changing time and circumstances [222]. Another alternative is to categorize individuals by ancestry, which can be defined geographically (e.g., Asian or sub-Saharan African), geopolitically (Japanese or Icelandic), or culturally (Lemba or Parsi) [223]. Genotyping Analysis of hundreds of polymorphic loci of globally distributed populations have shown the association of genetic variations with biogeographical ancestry [224]. In GWAS of large genotyped samples with admixture population structures, population stratification can arise when both the allele frequency and the distribution of a trait under study differ among subgroups of people included in the study. Unless corrected for, the population stratification can lead to excess false-positives [225]. Bioinformatics tools devised to correct for population stratification, such as EIGENSTRAT [225], STRUCTURE [226], fastStructure [227], ADMIXTURE [228], and AIPS [229] can be used to accurately validate self-reported ancestry or estimate unknown ancestry of cases. Using these tools to distinguish between population groups may be practical step until a standard set of race and ethnicity measures can be established.

The current lack of diversity in human genomic studies can be attributed to both scientific and logistical challenges, such as difficulty recruiting participants from minority populations, and unequal distribution of biomedical funding [218,230]. While the inclusion policy has the potential to help understand and eliminate health disparities, emphasizing the proportional recruitment of minority groups in NIH funded studies may not be the most effective way to achieve this goal. Critics argue that insufficient participation and underrepresented sub-samples of minority groups in genetic and genomic studies can limit statistical power required for robust subgroup analysis and reduce the accuracy and rigor of these studies. A better alternative may be to carry out targeted studies tailored directly to the minority population of interest, which can lead to greater ease of discovery [218]. A recent joint report aimed at enhancing cancer health disparities research offered recommendations that are relevant to the racial and ethnic disparity in CaP genomics. These recommendations include funding additional collaborative transdisciplinary studies focused on populations with unequal burdens of particular cancers, ensuring major initiatives (e.g., TCGA, Precision Medicine Initiative, and Cancer Moonshot Initiative) include sufficient representation of minority populations, developing and enhancing existing national biorepositories of solid cancers from underserved populations, and creating consortia that gather relevant biospecimen, clinical, data needed to conduct adequately powered hypothesis-driven health disparities research [230].

There are concerns that the use of racial or ethnic categories can lead to dangerous stereotyping in medical practice or send the harmful message to the broader public that distinctions between socially defined populations are genetically well established [231]. To allay these concerns, it is important to stress that the goal of ensuring adequate participation of minority racial and ethnic populations in cancer genomics research is to ensure that we do not miss finding rare gene alterations in these populations. These discoveries, when translated into clinical interventions will be used to treat the disease based on the gene alterations of the cancer, regardless of the race or ethnicity of the individual [213]. Therefore, increasing the participation of minority populations could help the discovery of rare driver gene mutations that enhance the accessibility of personalized medicine. This could prevent the cost of ineffective treatments, reduce morbidity, improve outcomes, and may contribute to overcoming the racial and ethnic disparity in CaP.

## Disclaimer

The views, opinions or assertions expressed in this article represent those of the authors and do not reflect the views or the official policy of the Department of Army, Navy or Air Force, Department of Defense, the Uniformed Services University of the Health Sciences or any other agency of the U.S. Government. The identification of specific products or scientific instrumentation is considered an integral part of the scientific endeavor and does not constitute endorsement or implied endorsement on the part of the author, DoD, or any component agency.

## Abbreviations

3C	chromosome conformation capture
4C	circularized chromosome conformation capture
AR	Androgen Receptor
CNAs	Copy Number Alterations
CPC-GENE	Canadian CaP Genome Network
CR-NEPC	castration resistant neuroendocrine prostate cancer
CRPC	castration resistant prostate adenocarcinoma
DDR	Damage Repair
DSB	double-strand break
EGFR	epidermal growth factor receptor
FA	Fanconi anemia
FISH	fluorescent in-situ hybridization
HBOC	hereditary breast and ovarian cancer
HBOC	ovarian cancer
HPC	hereditary Prostate Cancer
HR	homologous recombination
HR	homologous recombination
ICGC	International Cancer Genome Consortium
LS MAPK	Lynch Syndrome Mitogen-activated protein kinase
MATCH	Molecular Analysis for Therapy Choice
mCRPC	metastatic castrate resistant prostate cancer
MMR	Mismatch Repair
MSI	microsatellite instability
NCI	National Cancer Institute
NGS NIH	Next-Gen sequencing National Institutes of Health
NHEJ	Non-homologous end joining
NSCLC	non-small-cell lung cancer
OMB PARP PI3K	Office of Management and Budget Poly (ADP-ribose) polymerase Phosphatidylinositol 3- kinase
PCF	Prostate Cancer Foundation
SEER SNPs	Surveillance, Epidemiology, and End Results single-nucleotide polymorphisms
SSA	single-strand annealing
SU2C	Stand Up to Cancer
TCGA	The Cancer Genome Atlas
TSG	tumor suppressor genes
WES	Whole exome sequencing

## References

[1] Torre L.A., Bray F., Siegel R.L., Ferlay J., Lortet-Tieulent J., Jemal A. (2015). Global Cancer Statistics, 2012. CA Cancer J. Clin..

[2] Siegel R.L., Miller K.D., Jemal A. (2018). Cancer Statistics, 2018. CA Cancer J. Clin..

[3] DeSantis C.E., Siegel R.L., Sauer A.G., Miller K.D., Fedewa S.A., Alcaraz K.I., Jemal A. (2016). Cancer Statistics for African Americans, 2016: Progress and Opportunities in Reducing Racial Disparities. CA Cancer J. Clin..

[4] Chornokur G., Dalton K., Borysova M.E., Kumar N.B. (2011). Disparities at Presentation, Diagnosis, Treatment, and Survival in African American Men, Affected by Prostate Cancer. Prostate.

[5] Schwartz K., Powell I.J., Underwood W., George J., Yee C., Banerjee M. (2009). Interplay of Race, Socioeconomic Status, and Treatment on Survival of Patients with Prostate Cancer. Urology.

[6] Cheng I., Witte J.S., McClure L.A., Shema S.J., Cockburn M.G., John E.M., Clarke C.A. (2009). Socioeconomic Status and Prostate Cancer Incidence and Mortality Rates among the Diverse Population of California. Cancer Causes Control.

[7] Farrell J., Petrovics G., McLeod D.G., Srivastava S. (2013). Genetic and Molecular Differences in Prostate Carcinogenesis between African American and Caucasian American Men. Int. J. Mol. Sci..

[8] Hanna M.C., Go C., Roden C., Jones R.T., Pochanard P., Javed A.Y., Javed A., Mondal C., Palescandolo E., Van Hummelen P. (2013). Colorectal Cancers from Distinct Ancestral Populations Show Variations in Braf Mutation Frequency. PLoS ONE.

[9] Sun Y., Ren Y., Fang Z., Li C., Fang R., Gao B., Han X., Tian W., Pao W., Chen H. (2010). Lung Adenocarcinoma from East Asian Never-Smokers Is a Disease Largely Defined by Targetable Oncogenic Mutant Kinases. J. Clin. Oncol..

[10] Guda K., Veigl M.L., Varadan V., Nosrati A., Ravi L., Lutterbaugh J., Beard L., Willson J.K., Sedwick W.D., Wang Z.J. (2015). Novel Recurrently Mutated Genes in African American Colon Cancers. Proc. Natl. Acad. Sci. USA.

[11] Haber D.A., Bell D.W., Sordella R., Kwak E.L., Godin-Heymann N., Sharma S.V., Lynch T.J., Settleman J. (2005). Molecular Targeted Therapy of Lung Cancer: EGFR Mutations and Response to Egfr Inhibitors. Cold Spring Harb. Symp. Quant. Biol..

[12] Sanger F., Nicklen S., Coulson A.R. (1977). DNA Sequencing with Chain-Terminating Inhibitors. Proc. Natl. Acad. Sci. USA.

[13] Venter J.C., Adams M.D., Myers E.W., Li P.W., Mural R.J., Sutton G.G., Smith H.O., Yandell M., Evans C.A., Holt R.A. (2001). The Sequence of the Human Genome. Science.

[14] Lander E.S., Linton L.M., Birren B., Nusbaum C., Zody M.C., Baldwin J., Devon K., Dewar K., Doyle M., FitzHugh W. (2001). Initial Sequencing and Analysis of the Human Genome. Nature.

[15] Thorisson G.A., Smith A.V., Krishnan L., Stein L.D. (2005). The International Hapmap Project Web Site. Genome Res..

[16] Auton A., Brooks L.D., Durbin R.M., Garrison E.P., Kang H.M., Korbel J.O., Marchini J.L., McCarthy S., McVean G.A., Abecasis G.R. (2015). A Global Reference for Human Genetic Variation. Nature.

[17] Stranger B.E., Stahl E.A., Raj T. (2011). Progress and Promise of Genome-Wide Association Studies for Human Complex Trait Genetics. Genetics.

[18] Spratt D.E., Chan T., Waldron L., Speers C., Feng F.Y., Ogunwobi O.O., Osborne J.R. (2016). Racial/Ethnic Disparities in Genomic Sequencing. JAMA Oncol..

[19] Page W.F., Braun M.M., Partin A.W., Caporaso N., Walsh P. (1997). Heredity and Prostate Cancer: A Study of World War Ii Veteran Twins. Prostate.

[20] Ahlbom A., Lichtenstein P., Malmstrom H., Feychting M., Hemminki K., Pedersen N.L. (1997). Cancer in Twins: Genetic and Nongenetic Familial Risk Factors. J. Natl. Cancer Inst..

[21] Mucci L.A., Hjelmborg J.B., Harris J.R., Czene K., Havelick D.J., Scheike T., Graff R.E., Holst K., Moller S., Unger R.H. (2016). Familial Risk and Heritability of Cancer among Twins in Nordic Countries. JAMA.

[22] Albright F.S., Stephenson R.A., Agarwal N., Cannon-Albright L.A. (2017). Relative Risks for Lethal Prostate Cancer Based on Complete Family History of Prostate Cancer Death. Prostate.

[23] Whittemore A.S., Wu A.H., Kolonel L.N., John E.M., Gallagher R.P., Howe G.R., West D.W., Teh C.Z., Stamey T. (1995). Family History and Prostate Cancer Risk in Black, White, and Asian Men in the United States and Canada. Am. J. Epidemiol..

[24] Carter B.S., Beaty T.H., Steinberg G.D., Childs B., Walsh P.C. (1992). Mendelian Inheritance of Familial Prostate Cancer. Proc. Natl. Acad. Sci. USA.

[25] Gronberg H., Damber L., Damber J.E., Iselius L. (1997). Segregation Analysis of Prostate Cancer in Sweden: Support for Dominant Inheritance. Am. J. Epidemiol..

[26] MacInnis R.J., Antoniou A.C., Eeles R.A., Severi G., Guy M., McGuffog L., Hall A.L., O’Brien L.T., Wilkinson R.A., Dearnaley D.P. (2010). Prostate Cancer Segregation Analyses Using 4390 Families from UK and Australian Population-Based Studies. Genet. Epidemiol..

[27] Pakkanen S., Baffoe-Bonnie A.B., Matikainen M.P., Koivisto P.A., Tammela T.L., Deshmukh S., Ou L., Bailey-Wilson J.E., Schleutker J. (2007). Segregation Analysis of 1546 Prostate Cancer Families in Finland Shows Recessive Inheritance. Hum. Genet..

[28] Pilie P.G., Giri V.N., Cooney K.A. (2016). Hoxb13 and Other High Penetrant Genes for Prostate Cancer. Asian J. Androl..

[29] Ewing C.M., Ray A.M., Lange E.M., Zuhlke K.A., Robbins C.M., Tembe W.D., Wiley K.E., Isaacs S.D., Johng D., Wang Y. (2012). Germline Mutations in Hoxb13 and Prostate-Cancer Risk. N. Engl. J. Med..

[30] Pritchard C.C., Mateo J., Walsh M.F., De Sarkar N., Abida W., Beltran H., Garofalo A., Gulati R., Carreira S., Eeles R. (2016). Inherited DNA-Repair Gene Mutations in Men with Metastatic Prostate Cancer. N. Engl. J. Med..

[31] Nagy R., Sweet K., Eng C. (2004). Highly Penetrant Hereditary Cancer Syndromes. Oncogene.

[32] Simard J., Dumont M., Labuda D., Sinnett D., Meloche C., El-Alfy M., Berger L., Lees E., Labrie F., Tavtigian S.V. (2003). Prostate Cancer Susceptibility Genes: Lessons Learned and Challenges Posed. Endocr. Relat. Cancer.

[33] Sfanos K.S., De Marzo A.M. (2012). Prostate Cancer and Inflammation: The Evidence. Histopathology.

[34] Witte J.S. (2009). Prostate Cancer Genomics: Towards a New Understanding. Nat. Rev. Genet..

[35] Pomerantz M.M., Freedman M.L. (2010). Genetics of Prostate Cancer Risk. Mt. Sinai J. Med. N. Y..

[36] Hormozdiari F., Kichaev G., Yang W.Y., Pasaniuc B., Eskin E. (2015). Identification of Causal Genes for Complex Traits. Bioinformatics.

[37] Kote-Jarai Z., Easton D.F., Stanford J.L., Ostrander E.A., Schleutker J., Ingles S.A., Schaid D., Thibodeau S., Dork T., Neal D. (2008). Multiple Novel Prostate Cancer Predisposition Loci Confirmed by an International Study: The Practical Consortium. Cancer Epidemiol. Biomark. Prev..

[38] Al Olama A.A., Dadaev T., Hazelett D.J., Li Q., Leongamornlert D., Saunders E.J., Stephens S., Cieza-Borrella C., Whitmore I., Benlloch Garcia S. (2015). Multiple Novel Prostate Cancer Susceptibility Signals Identified by Fine-Mapping of Known Risk Loci among Europeans. Hum. Mol. Genet..

[39] Mancuso N., Rohland N., Rand K.A., Tandon A., Allen A., Quinque D., Mallick S., Li H., Stram A., Sheng X. (2016). The Contribution of Rare Variation to Prostate Cancer Heritability. Nat. Genet..

[40] Auer P.L., Lettre G. (2015). Rare Variant Association Studies: Considerations, Challenges and Opportunities. Genome Med..

[41] McCarthy S., Das S., Kretzschmar W., Delaneau O., Wood A.R., Teumer A., Kang H.M., Fuchsberger C., Danecek P., Sharp K. (2016). A Reference Panel of 64,976 Haplotypes for Genotype Imputation. Nat. Genet..

[42] Huang J., Howie B., McCarthy S., Memari Y., Walter K., Min J.L., Danecek P., Malerba G., Trabetti E., Zheng H.F. (2015). Improved Imputation of Low-Frequency and Rare Variants Using the UK10K Haplotype Reference Panel. Nat. Commun..

[43] Sud A., Kinnersley B., Houlston R.S. (2017). Genome-Wide Association Studies of Cancer: Current Insights and Future Perspectives. Nat. Rev. Cancer.

[44] Howie B.N., Donnelly P., Marchini J. (2009). A Flexible and Accurate Genotype Imputation Method for the Next Generation of Genome-Wide Association Studies. PLoS Genet..

[45] Amundadottir L.T., Sulem P., Gudmundsson J., Helgason A., Baker A., Agnarsson B.A., Sigurdsson A., Benediktsdottir K.R., Cazier J.B., Sainz J. (2006). A Common Variant Associated with Prostate Cancer in European and African Populations. Nat. Genet..

[46] Gudmundsson J., Sulem P., Manolescu A., Amundadottir L.T., Gudbjartsson D., Helgason A., Rafnar T., Bergthorsson J.T., Agnarsson B.A., Baker A. (2007). Genome-Wide Association Study Identifies a Second Prostate Cancer Susceptibility Variant at 8q24. Nat. Genet..

[47] Yeager M., Orr N., Hayes R.B., Jacobs K.B., Kraft P., Wacholder S., Minichiello M.J., Fearnhead P., Yu K., Chatterjee N. (2007). Genome-Wide Association Study of Prostate Cancer Identifies a Second Risk Locus at 8q24. Nat. Genet..

[48] Freedman M.L., Haiman C.A., Patterson N., McDonald G.J., Tandon A., Waliszewska A., Penney K., Steen R.G., Ardlie K., John E.M. (2006). Admixture Mapping Identifies 8q24 as a Prostate Cancer Risk Locus in African-American Men. Proc. Natl. Acad. Sci. USA.

[49] Schumacher F.R., Feigelson H.S., Cox D.G., Haiman C.A., Albanes D., Buring J., Calle E.E., Chanock S.J., Colditz G.A., Diver W.R. (2007). A Common 8q24 Variant in Prostate and Breast Cancer from a Large Nested Case-Control Study. Cancer Res..

[50] Haiman C.A., Patterson N., Freedman M.L., Myers S.R., Pike M.C., Waliszewska A., Neubauer J., Tandon A., Schirmer C., McDonald G.J. (2007). Multiple Regions within 8q24 Independently Affect Risk for Prostate Cancer. Nat. Genet..

[51] Haiman C.A., Chen G.K., Blot W.J., Strom S.S., Berndt S.I., Kittles R.A., Rybicki B.A., Isaacs W.B., Ingles S.A., Stanford J.L. (2011). Characterizing Genetic Risk at Known Prostate Cancer Susceptibility Loci in African Americans. PLoS Genet..

[52] Troutman S.M., Sissung T.M., Cropp C.D., Venzon D.J., Spencer S.D., Adesunloye B.A., Huang X., Karzai F.H., Price D.K., Figg W.D. (2012). Racial Disparities in the Association between Variants on 8q24 and Prostate Cancer: A Systematic Review and Meta-Analysis. Oncologist.

[53] Haiman C.A., Chen G.K., Blot W.J., Strom S.S., Berndt S.I., Kittles R.A., Rybicki B.A., Isaacs W.B., Ingles S.A., Stanford J.L. (2011). Genome-Wide Association Study of Prostate Cancer in Men of African Ancestry Identifies a Susceptibility Locus at 17q21. Nat. Genet..

[54] Taioli E., Sears V., Watson A., Flores-Obando R.E., Jackson M.D., Ukoli F.A., de Syllos Colus I.M., Fernandez P., McFarlane-Anderson N., Ostrander E.A. (2013). Polymorphisms in Cyp17 and Cyp3a4 and Prostate Cancer in Men of African Descent. Prostate.

[55] Whitman E.J., Pomerantz M., Chen Y., Chamberlin M.M., Furusato B., Gao C., Ali A., Ravindranath L., Dobi A., Sesterhenn I.A. (2010). Prostate Cancer Risk Allele Specific for African Descent Associates with Pathologic Stage at Prostatectomy. Cancer Epidemiol. Biomark. Prev..

[56] Koboldt D.C., Kanchi K.L., Gui B., Larson D.E., Fulton R.S., Isaacs W.B., Kraja A., Borecki I.B., Jia L., Wilson R.K. (2016). Rare Variation in Tet2 Is Associated with Clinically Relevant Prostate Carcinoma in African Americans. Cancer Epidemiol. Biomark. Prev..

[57] Eeles R.A., Olama A.A., Benlloch S., Saunders E.J., Leongamornlert D.A., Tymrakiewicz M., Ghoussaini M., Luccarini C., Dennis J., Jugurnauth-Little S. (2013). Identification of 23 New Prostate Cancer Susceptibility Loci Using the Icogs Custom Genotyping Array. Nat. Genet..

[58] Sampson J.N., Wheeler W.A., Yeager M., Panagiotou O., Wang Z., Berndt S.I., Lan Q., Abnet C.C., Amundadottir L.T., Figueroa J.D. (2015). Analysis of Heritability and Shared Heritability Based on Genome-Wide Association Studies for Thirteen Cancer Types. J. Natl. Cancer Inst..

[59] Al Olama A.A., Kote-Jarai Z., Berndt S.I., Conti D.V., Schumacher F., Han Y., Benlloch S., Hazelett D.J., Wang Z., Saunders E. (2014). A Meta-Analysis of 87,040 Individuals Identifies 23 New Susceptibility Loci for Prostate Cancer. Nat. Genet..

[60] Cheng I., Chen G.K., Nakagawa H., He J., Wan P., Laurie C.C., Shen J., Sheng X., Pooler L.C., Crenshaw A.T. (2012). Evaluating Genetic Risk for Prostate Cancer among Japanese and Latinos. Cancer Epidemiol. Biomark. Prev..

[61] Hoffmann T.J., Sakoda L.C., Shen L., Jorgenson E., Habel L.A., Liu J., Kvale M.N., Asgari M.M., Banda Y., Corley D. (2015). Imputation of the Rare Hoxb13 G84E Mutation and Cancer Risk in a Large Population-Based Cohort. PLoS Genet..

[62] Eeles R.A., Kote-Jarai Z., Al Olama A.A., Giles G.G., Guy M., Severi G., Muir K., Hopper J.L., Henderson B.E., Haiman C.A. (2009). Identification of Seven New Prostate Cancer Susceptibility Loci through a Genome-Wide Association Study. Nat. Genet..

[63] Nam R.K., Zhang W., Siminovitch K., Shlien A., Kattan M.W., Klotz L.H., Trachtenberg J., Lu Y., Zhang J., Yu C. (2011). New Variants at 10q26 and 15q21 Are Associated with Aggressive Prostate Cancer in a Genome-Wide Association Study from a Prostate Biopsy Screening Cohort. Cancer Biol. Ther..

[64] Kote-Jarai Z., Olama A.A., Giles G.G., Severi G., Schleutker J., Weischer M., Campa D., Riboli E., Key T., Gronberg H. (2011). Seven Prostate Cancer Susceptibility Loci Identified by a Multi-Stage Genome-Wide Association Study. Nat. Genet..

[65] Xu J., Kibel A.S., Hu J.J., Turner A.R., Pruett K., Zheng S.L., Sun J., Isaacs S.D., Wiley K.E., Kim S.T. (2009). Prostate Cancer Risk Associated Loci in African Americans. Cancer Epidemiol. Biomark. Prev..

[66] Hoffmann T.J., Van Den Eeden S.K., Sakoda L.C., Jorgenson E., Habel L.A., Graff R.E., Passarelli M.N., Cario C.L., Emami N.C., Chao C.R. (2015). A Large Multiethnic Genome-Wide Association Study of Prostate Cancer Identifies Novel Risk Variants and Substantial Ethnic Differences. Cancer Discov..

[67] Gusev A., Shi H., Kichaev G., Pomerantz M., Li F., Long H.W., Ingles S.A., Kittles R.A., Strom S.S., Rybicki B.A. (2016). Atlas of Prostate Cancer Heritability in European and African-American Men Pinpoints Tissue-Specific Regulation. Nat. Commun..

[68] Han Y., Signorello L.B., Strom S.S., Kittles R.A., Rybicki B.A., Stanford J.L., Goodman P.J., Berndt S.I., Carpten J., Casey G. (2015). Generalizability of Established Prostate Cancer Risk Variants in Men of African Ancestry. Int. J. Cancer.

[69] Han Y., Rand K.A., Hazelett D.J., Ingles S.A., Kittles R.A., Strom S.S., Rybicki B.A., Nemesure B., Isaacs W.B., Stanford J.L. (2016). Prostate Cancer Susceptibility in Men of African Ancestry at 8q24. J. Natl. Cancer Inst..

[70] Rand K.A., Rohland N., Tandon A., Stram A., Sheng X., Do R., Pasaniuc B., Allen A., Quinque D., Mallick S. (2016). Whole-Exome Sequencing of over 4100 Men of African Ancestry and Prostate Cancer Risk. Hum. Mol. Genet..

[71] Tan Y.C., Zeigler-Johnson C., Mittal R.D., Mandhani A., Mital B., Rebbeck T.R., Rennert H. (2008). Common 8q24 Sequence Variations Are Associated with Asian Indian Advanced Prostate Cancer Risk. Cancer Epidemiol. Biomark. Prev..

[72] Batra J., Lose F., Chambers S., Gardiner R.A., Aitken J., Yaxley J., Clements J.A., Spurdle A.B., Australian Prostate Cancer B. (2011). A Replication Study Examining Novel Common Single Nucleotide Polymorphisms Identified through a Prostate Cancer Genome-Wide Association Study in a Japanese Population. Am. J. Epidemiol..

[73] Xu J., Mo Z., Ye D., Wang M., Liu F., Jin G., Xu C., Wang X., Shao Q., Chen Z. (2012). Genome-Wide Association Study in Chinese Men Identifies Two New Prostate Cancer Risk Loci at 9q31.2 and 19q13.4. Nat. Genet..

[74] Na R., Liu F., Zhang P., Ye D., Xu C., Shao Q., Qi J., Wang X., Chen Z., Wang M. (2013). Evaluation of Reported Prostate Cancer Risk-Associated Snps from Genome-Wide Association Studies of Various Racial Populations in Chinese Men. Prostate.

[75] Marzec J., Mao X., Li M., Wang M., Feng N., Gou X., Wang G., Sun Z., Xu J., Xu H. (2016). A Genetic Study and Meta-Analysis of the Genetic Predisposition of Prostate Cancer in a Chinese Population. Oncotarget.

[76] MacArthur D.G., Manolio T.A., Dimmock D.P., Rehm H.L., Shendure J., Abecasis G.R., Adams D.R., Altman R.B., Antonarakis S.E., Ashley E.A. (2014). Guidelines for Investigating Causality of Sequence Variants in Human Disease. Nature.

[77] FitzGerald L.M., Raspin K., Marthick J.R., Field M.A., Malley R.C., Thomson R.J., Blackburn N.B., Banks A., Charlesworth J.C., Donovan S. (2017). Impact of the G84e Variant on Hoxb13 Gene and Protein Expression in Formalin-Fixed, Paraffin-Embedded Prostate Tumours. Sci. Rep..

[78] Choudhury A.D., Eeles R., Freedland S.J., Isaacs W.B., Pomerantz M.M., Schalken J.A., Tammela T.L., Visakorpi T. (2012). The Role of Genetic Markers in the Management of Prostate Cancer. Eur. Urol..

[79] Chen H., Ewing C.M., Zheng S., Grindedaal E.M., Cooney K.A., Wiley K., Djurovic S., Andreassen O.A., Axcrona K., Mills I.G. (2018). Genetic Factors Influencing Prostate Cancer Risk in Norwegian Men. Prostate.

[80] Giri V.N., Knudsen K.E., Kelly W.K., Abida W., Andriole G.L., Bangma C.H., Bekelman J.E., Benson M.C., Blanco A., Burnett A. (2018). Role of Genetic Testing for Inherited Prostate Cancer Risk: Philadelphia Prostate Cancer Consensus Conference 2017. J. Clin. Oncol..

[81] Chang B.L., Cramer S.D., Wiklund F., Isaacs S.D., Stevens V.L., Sun J., Smith S., Pruett K., Romero L.M., Wiley K.E. (2009). Fine Mapping Association Study and Functional Analysis Implicate a Snp in Msmb at 10q11 as a Causal Variant for Prostate Cancer Risk. Hum. Mol. Genet..

[82] Pomerantz M.M., Shrestha Y., Flavin R.J., Regan M.M., Penney K.L., Mucci L.A., Stampfer M.J., Hunter D.J., Chanock S.J., Schafer E.J. (2010). Analysis of the 10q11 Cancer Risk Locus Implicates Msmb and Ncoa4 in Human Prostate Tumorigenesis. PLoS Genet..

[83] Dias A., Kote-Jarai Z., Mikropoulos C., Eeles R. (2017). Prostate Cancer Germline Variations and Implications for Screening and Treatment. Cold Spring Harb. Perspect. Med..

[84] Stelloo S., Nevedomskaya E., Kim Y., Hoekman L., Bleijerveld O.B., Mirza T., Wessels L.F.A., van Weerden W.M., Altelaar A.F.M., Bergman A.M. (2018). Endogenous Androgen Receptor Proteomic Profiling Reveals Genomic Subcomplex Involved in Prostate Tumorigenesis. Oncogene.

[85] Rinckleb A.E., Surowy H.M., Luedeke M., Varga D., Schrader M., Hoegel J., Vogel W., Maier C. (2012). The Prostate Cancer Risk Locus at 10q11 Is Associated with DNA Repair Capacity. DNA Repair.

[86] El Gammal A.T., Bruchmann M., Zustin J., Isbarn H., Hellwinkel O.J., Kollermann J., Sauter G., Simon R., Wilczak W., Schwarz J. (2010). Chromosome 8p Deletions and 8q Gains Are Associated with Tumor Progression and Poor Prognosis in Prostate Cancer. Clin. Cancer Res..

[87] Gurel B., Iwata T., C M.K., Jenkins R.B., Lan F., Van Dang C., Hicks J.L., Morgan J., Cornish T.C., Sutcliffe S. (2008). Nuclear Myc Protein Overexpression Is an Early Alteration in Human Prostate Carcinogenesis. Mod. Pathol..

[88] Fromont G., Godet J., Peyret A., Irani J., Celhay O., Rozet F., Cathelineau X., Cussenot O. (2013). 8q24 Amplification Is Associated with Myc Expression and Prostate Cancer Progression and Is an Independent Predictor of Recurrence after Radical Prostatectomy. Hum. Pathol..

[89] Pomerantz M.M., Beckwith C.A., Regan M.M., Wyman S.K., Petrovics G., Chen Y., Hawksworth D.J., Schumacher F.R., Mucci L., Penney K.L. (2009). Evaluation of the 8q24 Prostate Cancer Risk Locus and Myc Expression. Cancer Res..

[90] Wasserman N.F., Aneas I., Nobrega M.A. (2010). An 8q24 Gene Desert Variant Associated with Prostate Cancer Risk Confers Differential in Vivo Activity to a Myc Enhancer. Genome Res..

[91] Pomerantz M.M., Ahmadiyeh N., Jia L., Herman P., Verzi M.P., Doddapaneni H., Beckwith C.A., Chan J.A., Hills A., Davis M. (2009). The 8q24 Cancer Risk Variant Rs6983267 Shows Long-Range Interaction with Myc in Colorectal Cancer. Nat. Genet..

[92] Ahmadiyeh N., Pomerantz M.M., Grisanzio C., Herman P., Jia L., Almendro V., He H.H., Brown M., Liu X.S., Davis M. (2010). 8q24 Prostate, Breast, and Colon Cancer Risk Loci Show Tissue-Specific Long-Range Interaction with Myc. Proc. Natl. Acad. Sci. USA.

[93] Cai M., Kim S., Wang K., Farnham P.J., Coetzee G.A., Lu W. (2016). 4c-Seq Revealed Long-Range Interactions of a Functional Enhancer at the 8q24 Prostate Cancer Risk Locus. Sci. Rep..

[94] Du M., Tillmans L., Gao J., Gao P., Yuan T., Dittmar R.L., Song W., Yang Y., Sahr N., Wang T. (2016). Chromatin Interactions and Candidate Genes at Ten Prostate Cancer Risk Loci. Sci. Rep..

[95] Ott J. (1991). Analysis of Human Genetic Linkage.

[96] Carpten J., Nupponen N., Isaacs S., Sood R., Robbins C., Xu J., Faruque M., Moses T., Ewing C., Gillanders E. (2002). Germline Mutations in the Ribonuclease L Gene in Families Showing Linkage with Hpc1. Nat. Genet..

[97] Tavtigian S.V., Simard J., Teng D.H., Abtin V., Baumgard M., Beck A., Camp N.J., Carillo A.R., Chen Y., Dayananth P. (2001). A Candidate Prostate Cancer Susceptibility Gene at Chromosome 17p. Nat. Genet..

[98] Xu J., Zheng S.L., Komiya A., Mychaleckyj J.C., Isaacs S.D., Hu J.J., Sterling D., Lange E.M., Hawkins G.A., Turner A. (2002). Germline Mutations and Sequence Variants of the Macrophage Scavenger Receptor 1 Gene Are Associated with Prostate Cancer Risk. Nat. Genet..

[99] Thibodeau S.N., French A.J., Roche P.C., Cunningham J.M., Tester D.J., Lindor N.M., Moslein G., Baker S.M., Liskay R.M., Burgart L.J. (1996). Altered Expression of Hmsh2 and Hmlh1 in Tumors with Microsatellite Instability and Genetic Alterations in Mismatch Repair Genes. Cancer Res..

[100] Leongamornlert D., Saunders E., Dadaev T., Tymrakiewicz M., Goh C., Jugurnauth-Little S., Kozarewa I., Fenwick K., Assiotis I., Barrowdale D. (2014). Frequent Germline Deleterious Mutations in DNA Repair Genes in Familial Prostate Cancer Cases Are Associated with Advanced Disease. Br. J. Cancer.

[101] Beebe-Dimmer J.L., Zuhlke K.A., Johnson A.M., Liesman D., Cooney K.A. (2018). Rare Germline Mutations in African American Men Diagnosed with Early-Onset Prostate Cancer. Prostate.

[102] Beebe-Dimmer J.L., Isaacs W.B., Zuhlke K.A., Yee C., Walsh P.C., Isaacs S.D., Johnson A.M., Ewing C.E., Humphreys E.B., Chowdhury W.H. (2014). Prevalence of the Hoxb13 G84e Prostate Cancer Risk Allele in Men Treated with Radical Prostatectomy. BJU Int..

[103] Na R., Zheng S.L., Han M., Yu H., Jiang D., Shah S., Ewing C.M., Zhang L., Novakovic K., Petkewicz J. (2017). Germline Mutations in Atm and Brca1/2 Distinguish Risk for Lethal and Indolent Prostate Cancer and Are Associated with Early Age at Death. Eur. Urol..

[104] Raymond V.M., Mukherjee B., Wang F., Huang S.C., Stoffel E.M., Kastrinos F., Syngal S., Cooney K.A., Gruber S.B. (2013). Elevated Risk of Prostate Cancer among Men with Lynch Syndrome. J. Clin. Oncol..

[105] Dominguez-Valentin M., Joost P., Therkildsen C., Jonsson M., Rambech E., Nilbert M. (2016). Frequent Mismatch-Repair Defects Link Prostate Cancer to Lynch Syndrome. BMC Urol..

[106] Guindalini R.S., Win A.K., Gulden C., Lindor N.M., Newcomb P.A., Haile R.W., Raymond V., Stoffel E., Hall M., Llor X. (2015). Mutation Spectrum and Risk of Colorectal Cancer in African American Families with Lynch Syndrome. Gastroenterology.

[107] Rebbeck T.R., Walker A.H., Zeigler-Johnson C., Weisburg S., Martin A.M., Nathanson K.L., Wein A.J., Malkowicz S.B. (2000). Association of Hpc2/Elac2 Genotypes and Prostate Cancer. Am. J. Hum. Genet..

[108] Severi G., Giles G.G., Southey M.C., Tesoriero A., Tilley W., Neufing P., Morris H., English D.R., McCredie M.R., Boyle P. (2003). Elac2/Hpc2 Polymorphisms, Prostate-Specific Antigen Levels, and Prostate Cancer. J. Natl. Cancer Inst..

[109] Xu B., Tong N., Li J.M., Zhang Z.D., Wu H.F. (2010). Elac2 Polymorphisms and Prostate Cancer Risk: A Meta-Analysis Based on 18 Case-Control Studies. Prostate Cancer Prostatic Dis..

[110] Robbins C.M., Hernandez W., Ahaghotu C., Bennett J., Hoke G., Mason T., Pettaway C.A., Vijayakumar S., Weinrich S., Furbert-Harris P. (2008). Association of Hpc2/Elac2 and Rnasel Non-Synonymous Variants with Prostate Cancer Risk in African American Familial and Sporadic Cases. Prostate.

[111] Casey G., Neville P.J., Plummer S.J., Xiang Y., Krumroy L.M., Klein E.A., Catalona W.J., Nupponen N., Carpten J.D., Trent J.M. (2002). Rnasel Arg462gln Variant Is Implicated in up to 13% of Prostate Cancer Cases. Nat. Genet..

[112] Shook S.J., Beuten J., Torkko K.C., Johnson-Pais T.L., Troyer D.A., Thompson I.M., Leach R.J. (2007). Association of Rnasel Variants with Prostate Cancer Risk in Hispanic Caucasians and African Americans. Clin. Cancer Res..

[113] Li H., Tai B.C. (2006). Rnasel Gene Polymorphisms and the Risk of Prostate Cancer: A Meta-Analysis. Clin. Cancer Res..

[114] Eeles R.A., Durocher F., Edwards S., Teare D., Badzioch M., Hamoudi R., Gill S., Biggs P., Dearnaley D., Ardern-Jones A. (1998). Linkage Analysis of Chromosome 1q Markers in 136 Prostate Cancer Families. The Cancer Research Campaign/British Prostate Group U.K. Familial Prostate Cancer Study Collaborators. Am. J. Hum. Genet..

[115] Wiklund F., Jonsson B.A., Brookes A.J., Stromqvist L., Adolfsson J., Emanuelsson M., Adami H.O., Augustsson-Balter K., Gronberg H. (2004). Genetic Analysis of the Rnasel Gene in Hereditary, Familial, and Sporadic Prostate Cancer. Clin. Cancer Res..

[116] Brown W.M., Lange E.M., Chen H., Zheng S.L., Chang B., Wiley K.E., Isaacs S.D., Walsh P.C., Isaacs W.B., Xu J. (2004). Hereditary Prostate Cancer in African American Families: Linkage Analysis Using Markers That Map to Five Candidate Susceptibility Loci. Br. J. Cancer.

[117] Powell I.J., Carpten J., Dunston G., Kittles R., Bennett J., Hoke G., Pettaway C., Weinrich S., Vijayakumar S., Ahaghotu C.A. (2001). African-American Heredity Prostate Cancer Study: A Model for Genetic Research. J. Natl. Med. Assoc..

[118] Xu J., Zheng S.L., Hawkins G.A., Faith D.A., Kelly B., Isaacs S.D., Wiley K.E., Chang B., Ewing C.M., Bujnovszky P. (2001). Linkage and Association Studies of Prostate Cancer Susceptibility: Evidence for Linkage at 8p22-23. Am. J. Hum. Genet..

[119] Wiklund F., Jonsson B.A., Goransson I., Bergh A., Gronberg H. (2003). Linkage Analysis of Prostate Cancer Susceptibility: Confirmation of Linkage at 8p22-23. Hum. Genet..

[120] Sun J., Hsu F.C., Turner A.R., Zheng S.L., Chang B.L., Liu W., Isaacs W.B., Xu J. (2006). Meta-Analysis of Association of Rare Mutations and Common Sequence Variants in the Msr1 Gene and Prostate Cancer Risk. Prostate.

[121] Beuten J., Gelfond J.A., Franke J.L., Shook S., Johnson-Pais T.L., Thompson I.M., Leach R.J. (2010). Single and Multivariate Associations of Msr1, Elac2, and Rnasel with Prostate Cancer in an Ethnic Diverse Cohort of Men. Cancer Epidemiol. Biomark. Prev..

[122] Dhanasekaran S.M., Barrette T.R., Ghosh D., Shah R., Varambally S., Kurachi K., Pienta K.J., Rubin M.A., Chinnaiyan A.M. (2001). Delineation of Prognostic Biomarkers in Prostate Cancer. Nature.

[123] Norris J.D., Chang C.Y., Wittmann B.M., Kunder R.S., Cui H., Fan D., Joseph J.D., McDonnell D.P. (2009). The Homeodomain Protein Hoxb13 Regulates the Cellular Response to Androgens. Mol. Cell.

[124] Pomerantz M.M., Li F., Takeda D.Y., Lenci R., Chonkar A., Chabot M., Cejas P., Vazquez F., Cook J., Shivdasani R.A. (2015). The Androgen Receptor Cistrome Is Extensively Reprogrammed in Human Prostate Tumorigenesis. Nat. Genet..

[125] Xu J., Lange E.M., Lu L., Zheng S.L., Wang Z., Thibodeau S.N., Cannon-Albright L.A., Teerlink C.C., Camp N.J., Johnson A.M. (2013). Hoxb13 Is a Susceptibility Gene for Prostate Cancer: Results from the International Consortium for Prostate Cancer Genetics (Icpcg). Hum. Genet..

[126] Witte J.S., Mefford J., Plummer S.J., Liu J., Cheng I., Klein E.A., Rybicki B.A., Casey G. (2013). Hoxb13 Mutation and Prostate Cancer: Studies of Siblings and Aggressive Disease. Cancer Epidemiol. Biomark. Prev..

[127] Cai Q., Wang X., Li X., Gong R., Guo X., Tang Y., Yang K., Niu Y., Zhao Y. (2015). Germline Hoxb13 P.Gly84glu Mutation and Cancer Susceptibility: A Pooled Analysis of 25 Epidemiological Studies with 145,257 Participates. Oncotarget.

[128] Maia S., Cardoso M., Pinto P., Pinheiro M., Santos C., Peixoto A., Bento M.J., Oliveira J., Henrique R., Jeronimo C. (2015). Identification of Two Novel Hoxb13 Germline Mutations in Portuguese Prostate Cancer Patients. PLoS ONE.

[129] Laitinen V.H., Wahlfors T., Saaristo L., Rantapero T., Pelttari L.M., Kilpivaara O., Laasanen S.L., Kallioniemi A., Nevanlinna H., Aaltonen L. (2013). Hoxb13 G84e Mutation in Finland: Population-Based Analysis of Prostate, Breast, and Colorectal Cancer Risk. Cancer Epidemiol. Biomark. Prev..

[130] Lin X., Qu L., Chen Z., Xu C., Ye D., Shao Q., Wang X., Qi J., Chen Z., Zhou F. (2013). A Novel Germline Mutation in Hoxb13 Is Associated with Prostate Cancer Risk in Chinese Men. Prostate.

[131] Petrucelli N., Daly M.B., Feldman G.L. (2010). Hereditary Breast and Ovarian Cancer Due to Mutations in Brca1 and Brca2. Genet. Med..

[132] Roy R., Chun J., Powell S.N. (2011). Brca1 and Brca2: Different Roles in a Common Pathway of Genome Protection. Nat. Rev. Cancer.

[133] Deng C.X. (2006). Brca1: Cell Cycle Checkpoint, Genetic Instability, DNA Damage Response and Cancer Evolution. Nucleic Acids Res..

[134] Moynahan M.E., Pierce A.J., Jasin M. (2001). Brca2 Is Required for Homology-Directed Repair of Chromosomal Breaks. Mol. Cell.

[135] Eeles R., Goh C., Castro E., Bancroft E., Guy M., Al Olama A.A., Easton D., Kote-Jarai Z. (2014). The Genetic Epidemiology of Prostate Cancer and Its Clinical Implications. Nat. Rev. Urol..

[136] Li D., Kumaraswamy E., Harlan-Williams L.M., Jensen R.A. (2013). The Role of Brca1 and Brca2 in Prostate Cancer. Front. Biosci..

[137] Thompson D., Easton D.F., Breast Cancer Linkage C. (2002). Cancer Incidence in Brca1 Mutation Carriers. J. Natl. Cancer Inst..

[138] Breast Cancer Linkage Consortium (1999). Cancer Risks in Brca2 Mutation Carriers. J. Natl. Cancer Inst..

[139] Douglas J.A., Levin A.M., Zuhlke K.A., Ray A.M., Johnson G.R., Lange E.M., Wood D.P., Cooney K.A. (2007). Common Variation in the Brca1 Gene and Prostate Cancer Risk. Cancer Epidemiol. Biomark. Prev..

[140] Struewing J.P., Hartge P., Wacholder S., Baker S.M., Berlin M., McAdams M., Timmerman M.M., Brody L.C., Tucker M.A. (1997). The Risk of Cancer Associated with Specific Mutations of Brca1 and Brca2 among Ashkenazi Jews. N. Engl. J. Med..

[141] Kirchhoff T., Kauff N.D., Mitra N., Nafa K., Huang H., Palmer C., Gulati T., Wadsworth E., Donat S., Robson M.E. (2004). Brca Mutations and Risk of Prostate Cancer in Ashkenazi Jews. Clin. Cancer Res..

[142] Agalliu I., Gern R., Leanza S., Burk R.D. (2009). Associations of High-Grade Prostate Cancer with Brca1 and Brca2 Founder Mutations. Clin. Cancer Res..

[143] John E.M., Miron A., Gong G., Phipps A.I., Felberg A., Li F.P., West D.W., Whittemore A.S. (2007). Prevalence of Pathogenic Brca1 Mutation Carriers in 5 Us Racial/Ethnic Groups. JAMA.

[144] Sigurdsson S., Thorlacius S., Tomasson J., Tryggvadottir L., Benediktsdottir K., Eyfjord J.E., Jonsson E. (1997). Brca2 Mutation in Icelandic Prostate Cancer Patients. J. Mol. Med..

[145] Gayther S.A., de Foy K.A., Harrington P., Pharoah P., Dunsmuir W.D., Edwards S.M., Gillett C., Ardern-Jones A., Dearnaley D.P., Easton D.F. (2000). The Frequency of Germ-Line Mutations in the Breast Cancer Predisposition Genes Brca1 and Brca2 in Familial Prostate Cancer. The Cancer Research Campaign/British Prostate Group United Kingdom Familial Prostate Cancer Study Collaborators. Cancer Res..

[146] Gronberg H., Ahman A.K., Emanuelsson M., Bergh A., Damber J.E., Borg A. (2001). Brca2 Mutation in a Family with Hereditary Prostate Cancer. Genes Chromosomes Cancer.

[147] Bancroft E.K., Page E.C., Castro E., Lilja H., Vickers A., Sjoberg D., Assel M., Foster C.S., Mitchell G., Drew K. (2014). Targeted Prostate Cancer Screening in Brca1 and Brca2 Mutation Carriers: Results from the Initial Screening Round of the Impact Study. Eur. Urol..

[148] Akbari M.R., Wallis C.J., Toi A., Trachtenberg J., Sun P., Narod S.A., Nam R.K. (2014). The Impact of a Brca2 Mutation on Mortality from Screen-Detected Prostate Cancer. Br. J. Cancer.

[149] Leao R.R.N., Price A.J., James Hamilton R. (2017). Germline Brca Mutation in Male Carriers-Ripe for Precision Oncology?. Prostate Cancer Prostatic Dis..

[150] Grindedal E.M., Moller P., Eeles R., Stormorken A.T., Bowitz-Lothe I.M., Landro S.M., Clark N., Kvale R., Shanley S., Maehle L. (2009). Germ-Line Mutations in Mismatch Repair Genes Associated with Prostate Cancer. Cancer Epidemiol. Biomark. Prev..

[151] Win A.K., Lindor N.M., Young J.P., Macrae F.A., Young G.P., Williamson E., Parry S., Goldblatt J., Lipton L., Winship I. (2012). Risks of Primary Extracolonic Cancers Following Colorectal Cancer in Lynch Syndrome. J. Natl. Cancer Inst..

[152] Bauer C.M., Ray A.M., Halstead-Nussloch B.A., Dekker R.G., Raymond V.M., Gruber S.B., Cooney K.A. (2011). Hereditary Prostate Cancer as a Feature of Lynch Syndrome. Fam. Cancer.

[153] Langeberg W.J., Kwon E.M., Koopmeiners J.S., Ostrander E.A., Stanford J.L. (2010). Population-Based Study of the Association of Variants in Mismatch Repair Genes with Prostate Cancer Risk and Outcomes. Cancer Epidemiol. Biomark. Prev..

[154] Dong X., Wang L., Taniguchi K., Wang X., Cunningham J.M., McDonnell S.K., Qian C., Marks A.F., Slager S.L., Peterson B.J. (2003). Mutations in Chek2 Associated with Prostate Cancer Risk. Am. J. Hum. Genet..

[155] Seppala E.H., Ikonen T., Mononen N., Autio V., Rokman A., Matikainen M.P., Tammela T.L., Schleutker J. (2003). Chek2 Variants Associate with Hereditary Prostate Cancer. Br. J. Cancer.

[156] Angele S., Falconer A., Edwards S.M., Dork T., Bremer M., Moullan N., Chapot B., Muir K., Houlston R., Norman A.R. (2004). Atm Polymorphisms as Risk Factors for Prostate Cancer Development. Br. J. Cancer.

[157] Blackford A.N., Jackson S.P. (2017). Atm, Atr, and DNA-Pk: The Trinity at the Heart of the DNA Damage Response. Mol. Cell.

[158] Robinson D., Van Allen E.M., Wu Y.M., Schultz N., Lonigro R.J., Mosquera J.M., Montgomery B., Taplin M.E., Pritchard C.C., Attard G. (2015). Integrative Clinical Genomics of Advanced Prostate Cancer. Cell.

[159] Taylor B.S., Schultz N., Hieronymus H., Gopalan A., Xiao Y., Carver B.S., Arora V.K., Kaushik P., Cerami E., Reva B. (2010). Integrative Genomic Profiling of Human Prostate Cancer. Cancer Cell.

[160] Holcomb I.N., Young J.M., Coleman I.M., Salari K., Grove D.I., Hsu L., True L.D., Roudier M.P., Morrissey C.M., Higano C.S. (2009). Comparative Analyses of Chromosome Alterations in Soft-Tissue Metastases within and across Patients with Castration-Resistant Prostate Cancer. Cancer Res..

[161] Robbins C.M., Tembe W.A., Baker A., Sinari S., Moses T.Y., Beckstrom-Sternberg S., Beckstrom-Sternberg J., Barrett M., Long J., Chinnaiyan A. (2011). Copy Number and Targeted Mutational Analysis Reveals Novel Somatic Events in Metastatic Prostate Tumors. Genome Res..

[162] Kan Z., Jaiswal B.S., Stinson J., Janakiraman V., Bhatt D., Stern H.M., Yue P., Haverty P.M., Bourgon R., Zheng J. (2010). Diverse Somatic Mutation Patterns and Pathway Alterations in Human Cancers. Nature.

[163] Dong J.T. (2001). Chromosomal Deletions and Tumor Suppressor Genes in Prostate Cancer. Cancer Metastasis Rev..

[164] Nupponen N.N., Visakorpi T. (2000). Molecular Cytogenetics of Prostate Cancer. Microsc. Res. Tech..

[165] Ren G., Liu X., Mao X., Zhang Y., Stankiewicz E., Hylands L., Song R., Berney D.M., Clark J., Cooper C. (2012). Identification of Frequent Braf Copy Number Gain and Alterations of Raf Genes in Chinese Prostate Cancer. Genes Chromosomes Cancer.

[166] Petrovics G., Liu A., Shaheduzzaman S., Furusato B., Sun C., Chen Y., Nau M., Ravindranath L., Chen Y., Dobi A. (2005). Frequent Overexpression of Ets-Related Gene-1 (Erg1) in Prostate Cancer Transcriptome. Oncogene.

[167] Tomlins S.A., Rhodes D.R., Perner S., Dhanasekaran S.M., Mehra R., Sun X.W., Varambally S., Cao X., Tchinda J., Kuefer R. (2005). Recurrent Fusion of Tmprss2 and Ets Transcription Factor Genes in Prostate Cancer. Science.

[168] Hudson T.J., Anderson W., Artez A., Barker A.D., Bell C., Bernabe R.R., Bhan M.K., Calvo F., Eerola I., International Cancer Genome Consortium (2010). International Network of Cancer Genome Projects. Nature.

[169] Tomczak K., Czerwinska P., Wiznerowicz M. (2015). The Cancer Genome Atlas (Tcga): An Immeasurable Source of Knowledge. Contemp. Oncol..

[170] Berger M.F., Lawrence M.S., Demichelis F., Drier Y., Cibulskis K., Sivachenko A.Y., Sboner A., Esgueva R., Pflueger D., Sougnez C. (2011). The Genomic Complexity of Primary Human Prostate Cancer. Nature.

[171] Barbieri C.E., Baca S.C., Lawrence M.S., Demichelis F., Blattner M., Theurillat J.P., White T.A., Stojanov P., Van Allen E., Stransky N. (2012). Exome Sequencing Identifies Recurrent *SPOP*, *FOXA1* and *MED12* Mutations in Prostate Cancer. Nat. Genet..

[172] Baca S.C., Prandi D., Lawrence M.S., Mosquera J.M., Romanel A., Drier Y., Park K., Kitabayashi N., MacDonald T.Y., Ghandi M. (2013). Punctuated Evolution of Prostate Cancer Genomes. Cell.

[173] The Cancer Genome Atlas Research Network (2015). The Molecular Taxonomy of Primary Prostate Cancer. Cell.

[174] Fraser M., Sabelnykova V.Y., Yamaguchi T.N., Heisler L.E., Livingstone J., Huang V., Shiah Y.J., Yousif F., Lin X., Masella A.P. (2017). Genomic Hallmarks of Localized, Non-Indolent Prostate Cancer. Nature.

[175] Alexandrov L.B., Nik-Zainal S., Wedge D.C., Aparicio S.A., Behjati S., Biankin A.V., Bignell G.R., Bolli N., Borg A., Borresen-Dale A.L. (2013). Signatures of Mutational Processes in Human Cancer. Nature.

[176] Korbel J.O., Campbell P.J. (2013). Criteria for Inference of Chromothripsis in Cancer Genomes. Cell.

[177] Khani F., Mosquera J.M., Park K., Blattner M., O’Reilly C., MacDonald T.Y., Chen Z., Srivastava A., Tewari A.K., Barbieri C.E. (2014). Evidence for Molecular Differences in Prostate Cancer between African American and Caucasian Men. Clin. Cancer Res..

[178] Magi-Galluzzi C., Tsusuki T., Elson P., Simmerman K., LaFargue C., Esgueva R., Klein E., Rubin M.A., Zhou M. (2011). Tmprss2-Erg Gene Fusion Prevalence and Class Are Significantly Different in Prostate Cancer of Caucasian, African-American and Japanese Patients. Prostate.

[179] Rosen P., Pfister D., Young D., Petrovics G., Chen Y., Cullen J., Bohm D., Perner S., Dobi A., McLeod D.G. (2012). Differences in Frequency of Erg Oncoprotein Expression between Index Tumors of Caucasian and African American Patients with Prostate Cancer. Urology.

[180] Petrovics G., Li H., Stumpel T., Tan S.H., Young D., Katta S., Li Q., Ying K., Klocke B., Ravindranath L. (2015). A Novel Genomic Alteration of Lsamp Associates with Aggressive Prostate Cancer in African American Men. EBioMedicine.

[181] Barøy T., Kresse S.H., Skarn M., Stabell M., Castro R., Lauvrak S., Llombart-Bosch A., Myklebost O., Meza-Zepeda L.A. (2014). Reexpression of Lsamp Inhibits Tumor Growth in a Preclinical Osteosarcoma Model. Mol. Cancer.

[182] Chen J., Lui W.O., Vos M.D., Clark G.J., Takahashi M., Schoumans J., Khoo S.K., Petillo D., Lavery T., Sugimura J. (2003). The T(1;3) Breakpoint-Spanning Genes Lsamp and Nore1 Are Involved in Clear Cell Renal Cell Carcinomas. Cancer Cell.

[183] Ren S., Wei G.H., Liu D., Wang L., Hou Y., Zhu S., Peng L., Zhang Q., Cheng Y., Su H. (2017). Whole-Genome and Transcriptome Sequencing of Prostate Cancer Identify New Genetic Alterations Driving Disease Progression. Eur. Urol..

[184] Blattner M., Lee D.J., O’Reilly C., Park K., MacDonald T.Y., Khani F., Turner K.R., Chiu Y.L., Wild P.J., Dolgalev I. (2014). *SPOP* Mutations in Prostate Cancer across Demographically Diverse Patient Cohorts. Neoplasia.

[185] Grasso C.S., Wu Y.M., Robinson D.R., Cao X., Dhanasekaran S.M., Khan A.P., Quist M.J., Jing X., Lonigro R.J., Brenner J.C. (2012). The Mutational Landscape of Lethal Castration-Resistant Prostate Cancer. Nature.

[186] Liu W., Lindberg J., Sui G., Luo J., Egevad L., Li T., Xie C., Wan M., Kim S.T., Wang Z. (2012). Identification of Novel Chd1-Associated Collaborative Alterations of Genomic Structure and Functional Assessment of Chd1 in Prostate Cancer. Oncogene.

[187] Rodrigues L.U., Rider L., Nieto C., Romero L., Karimpour-Fard A., Loda M., Lucia M.S., Wu M., Shi L., Cimic A. (2015). Coordinate Loss of Map3k7 and Chd1 Promotes Aggressive Prostate Cancer. Cancer Res..

[188] Kari V., Mansour W.Y., Raul S.K., Baumgart S.J., Mund A., Grade M., Sirma H., Simon R., Will H., Dobbelstein M. (2016). Loss of Chd1 Causes DNA Repair Defects and Enhances Prostate Cancer Therapeutic Responsiveness. EMBO Rep..

[189] Shenoy T.R., Boysen G., Wang M.Y., Xu Q.Z., Guo W., Koh F.M., Wang C., Zhang L.Z., Wang Y., Gil V. (2017). Chd1 Loss Sensitizes Prostate Cancer to DNA Damaging Therapy by Promoting Error-Prone Double-Strand Break Repair. Ann. Oncol..

[190] Lindquist K.J., Paris P.L., Hoffmann T.J., Cardin N.J., Kazma R., Mefford J.A., Simko J.P., Ngo V., Chen Y., Levin A.M. (2016). Mutational Landscape of Aggressive Prostate Tumors in African American Men. Cancer Res..

[191] Schulz W.A., Elo J.P., Florl A.R., Pennanen S., Santourlidis S., Engers R., Buchardt M., Seifert H.H., Visakorpi T. (2002). Genomewide DNA Hypomethylation Is Associated with Alterations on Chromosome 8 in Prostate Carcinoma. Genes Chromosomes Cancer.

[192] Huang F.W., Mosquera J.M., Garofalo A., Oh C., Baco M., Amin-Mansour A., Rabasha B., Bahl S., Mullane S.A., Robinson B.D. (2017). Exome Sequencing of African-American Prostate Cancer Reveals Loss-of-Function Erf Mutations. Cancer Discov..

[193] Kumar A., White T.A., MacKenzie A.P., Clegg N., Lee C., Dumpit R.F., Coleman I., Ng S.B., Salipante S.J., Rieder M.J. (2011). Exome Sequencing Identifies a Spectrum of Mutation Frequencies in Advanced and Lethal Prostate Cancers. Proc. Natl. Acad. Sci. USA.

[194] Pritchard C.C., Morrissey C., Kumar A., Zhang X., Smith C., Coleman I., Salipante S.J., Milbank J., Yu M., Grady W.M. (2014). Complex Msh2 and Msh6 Mutations in Hypermutated Microsatellite Unstable Advanced Prostate Cancer. Nat. Commun..

[195] Kumar A., Coleman I., Morrissey C., Zhang X., True L.D., Gulati R., Etzioni R., Bolouri H., Montgomery B., White T. (2016). Substantial Interindividual and Limited Intraindividual Genomic Diversity among Tumors from Men with Metastatic Prostate Cancer. Nat. Med..

[196] Watson P.A., Arora V.K., Sawyers C.L. (2015). Emerging Mechanisms of Resistance to Androgen Receptor Inhibitors in Prostate Cancer. Nat. Rev. Cancer.

[197] Boudadi K., Antonarakis E.S. (2016). Resistance to Novel Antiandrogen Therapies in Metastatic Castration-Resistant Prostate Cancer. Clin. Med. Insights Oncol..

[198] Beltran H., Antonarakis E.S., Morris M.J., Attard G. (2016). Emerging Molecular Biomarkers in Advanced Prostate Cancer: Translation to the Clinic. Am. Soc. Clin. Oncol. Educ. Book.

[199] Abida W., Armenia J., Gopalan A., Brennan R., Walsh M., Barron D., Danila D., Rathkopf D., Morris M., Slovin S. (2017). Prospective Genomic Profiling of Prostate Cancer across Disease States Reveals Germline and Somatic Alterations That May Affect Clinical Decision Making. JCO Precis. Oncol..

[200] Romanel A., Garritano S., Stringa B., Blattner M., Dalfovo D., Chakravarty D., Soong D., Cotter K.A., Petris G., Dhingra P. (2017). Inherited Determinants of Early Recurrent Somatic Mutations in Prostate Cancer. Nat. Commun..

[201] Wei L., Wang J., Lampert E., Schlanger S., DePriest A.D., Hu Q., Gomez E.C., Murakam M., Glenn S.T., Conroy J. (2017). Intratumoral and Intertumoral Genomic Heterogeneity of Multifocal Localized Prostate Cancer Impacts Molecular Classifications and Genomic Prognosticators. Eur. Urol..

[202] Banks P., Xu W., Murphy D., James P., Sandhu S. (2017). Relevance of DNA Damage Repair in the Management of Prostate Cancer. Curr. Probl. Cancer.

[203] Mateo J., Boysen G., Barbieri C.E., Bryant H.E., Castro E., Nelson P.S., Olmos D., Pritchard C.C., Rubin M.A., de Bono J.S. (2017). DNA Repair in Prostate Cancer: Biology and Clinical Implications. Eur. Urol..

[204] Fong P.C., Yap T.A., Boss D.S., Carden C.P., Mergui-Roelvink M., Gourley C., De Greve J., Lubinski J., Shanley S., Messiou C. (2010). Poly(Adp)-Ribose Polymerase Inhibition: Frequent Durable Responses in Brca Carrier Ovarian Cancer Correlating with Platinum-Free Interval. J. Clin. Oncol..

[205] Mateo J., Carreira S., Sandhu S., Miranda S., Mossop H., Perez-Lopez R., Nava Rodrigues D., Robinson D., Omlin A., Tunariu N. (2015). DNA-Repair Defects and Olaparib in Metastatic Prostate Cancer. N. Engl. J. Med..

[206] Beltran H., Eng K., Mosquera J.M., Sigaras A., Romanel A., Rennert H., Kossai M., Pauli C., Faltas B., Fontugne J. (2015). Whole-Exome Sequencing of Metastatic Cancer and Biomarkers of Treatment Response. JAMA Oncol..

[207] Cheng H.H., Pritchard C.C., Boyd T., Nelson P.S., Montgomery B. (2016). Biallelic Inactivation of Brca2 in Platinum-Sensitive Metastatic Castration-Resistant Prostate Cancer. Eur. Urol..

[208] Mouw K.W., Goldberg M.S., Konstantinopoulos P.A., D’Andrea A.D. (2017). DNA Damage and Repair Biomarkers of Immunotherapy Response. Cancer Discov..

[209] Le D.T., Durham J.N., Smith K.N., Wang H., Bartlett B.R., Aulakh L.K., Lu S., Kemberling H., Wilt C., Luber B.S. (2017). Mismatch Repair Deficiency Predicts Response of Solid Tumors to Pd-1 Blockade. Science.

[210] Mateo J., Ganji G., Lemech C., Burris H.A., Han S.W., Swales K., Decordova S., DeYoung M.P., Smith D.A., Kalyana-Sundaram S. (2017). A First-Time-in-Human Study of Gsk2636771, a Phosphoinositide 3 Kinase Beta-Selective Inhibitor, in Patients with Advanced Solid Tumors. Clin. Cancer Res..

[211] Crumbaker M., Khoja L., Joshua A.M. (2017). AR Signaling and the Pi3k Pathway in Prostate Cancer. Cancers.

[212] Redig A.J., Janne P.A. (2015). Basket Trials and the Evolution of Clinical Trial Design in an Era of Genomic Medicine. J. Clin. Oncol..

[213] Schmidt K.T., Chau C.H., Price D.K., Figg W.D. (2016). Precision Oncology Medicine: The Clinical Relevance of Patient-Specific Biomarkers Used to Optimize Cancer Treatment. J. Clin. Pharmacol..

[214] Mathieson I., Reich D. (2017). Differences in the Rare Variant Spectrum among Human Populations. PLoS Genet..

[215] Lawrence M.S., Stojanov P., Mermel C.H., Robinson J.T., Garraway L.A., Golub T.R., Meyerson M., Gabriel S.B., Lander E.S., Getz G. (2014). Discovery and Saturation Analysis of Cancer Genes across 21 Tumour Types. Nature.

[216] Haga S.B. (2010). Impact of Limited Population Diversity of Genome-Wide Association Studies. Genet. Med..

[217] National Institutes of Health Nih Policy and Guidelines on the Inclusion of Women and Minorities as Subjects in Clinical Research—Amended 28 November 2017. https://grants.nih.gov/grants/funding/women_min/guidelines.htm.

[218] Knerr S., Wayman D., Bonham V.L. (2011). Inclusion of Racial and Ethnic Minorities in Genetic Research: Advance the Spirit by Changing the Rules?. J. Law Med. Ethics.

[219] Patten E. (2015). Who Is Multiracial? Depends on How You Ask.

[220] Bamshad M., Wooding S.P. (2003). Signatures of Natural Selection in the Human Genome. Nat. Rev. Genet..

[221] Baharian S., Barakatt M., Gignoux C.R., Shringarpure S., Errington J., Blot W.J., Bustamante C.D., Kenny E.E., Williams S.M., Aldrich M.C. (2016). The Great Migration and African-American Genomic Diversity. PLoS Genet..

[222] Perez A.D., Hirschman C. (2009). The Changing Racial and Ethnic Composition of the Us Population: Emerging American Identities. Popul. Dev. Rev..

[223] Elliott C., Brodwin P. (2002). Identity and Genetic Ancestry Tracing. BMJ.

[224] Bamshad M.J., Wooding S., Watkins W.S., Ostler C.T., Batzer M.A., Jorde L.B. (2003). Human Population Genetic Structure and Inference of Group Membership. Am. J. Hum. Genet..

[225] Price A.L., Patterson N.J., Plenge R.M., Weinblatt M.E., Shadick N.A., Reich D. (2006). Principal Components Analysis Corrects for Stratification in Genome-Wide Association Studies. Nat. Genet..

[226] Pritchard J.K., Stephens M., Donnelly P. (2000). Inference of Population Structure Using Multilocus Genotype Data. Genetics.

[227] Raj A., Stephens M., Pritchard J.K. (2014). Faststructure: Variational Inference of Population Structure in Large Snp Data Sets. Genetics.

[228] Alexander D.H., Lange K. (2011). Enhancements to the Admixture Algorithm for Individual Ancestry Estimation. BMC Bioinf..

[229] Byun J., Han Y., Gorlov I.P., Busam J.A., Seldin M.F., Amos C.I. (2017). Ancestry Inference Using Principal Component Analysis and Spatial Analysis: A Distance-Based Analysis to Account for Population Substructure. BMC Genom..

[230] Polite B.N., Adams-Campbell L.L., Brawley O.W., Bickell N., Carethers J.M., Flowers C.R., Foti M., Gomez S.L., Griggs J.J., Lathan C.S. (2017). Charting the Future of Cancer Health Disparities Research: A Position Statement from the American Association for Cancer Research, the American Cancer Society, the American Society of Clinical Oncology, and the National Cancer Institute. J. Clin. Oncol..

[231] Race Ethnicity Genetics Working Group (2005). The Use of Racial, Ethnic, and Ancestral Categories in Human Genetics Research. Am. J. Hum. Genet..

